# The Mechanism of Citrus Host Defense Response Repression at Early Stages of Infection by Feeding of *Diaphorina citri* Transmitting *Candidatus* Liberibacter asiaticus

**DOI:** 10.3389/fpls.2021.635153

**Published:** 2021-06-08

**Authors:** Xu Wei, Amany Mira, Qibin Yu, Fred G. Gmitter

**Affiliations:** ^1^Citrus Research and Education Center, University of Florida, Lake Alfred, FL, United States; ^2^College of Horticulture and Landscape, Southwest University, Chongqing, China; ^3^Department of Horticulture, Faculty of Agriculture, Tanta University, Tanta, Egypt

**Keywords:** Huanglongbing, *Candidatus* Liberibacter asiaticus, Asian citrus psyllid, RNA-seq, ATP, repressed defense

## Abstract

Citrus Huanglongbing (HLB) is the most devastating disease of citrus, presumably caused by “*Candidatus* Liberibacter asiaticus” (*Ca*Las). Although transcriptomic profiling of HLB-affected citrus plants has been studied extensively, the initial steps in pathogenesis have not been fully understood. In this study, RNA sequencing (RNA-seq) was used to compare very early transcriptional changes in the response of Valencia sweet orange (VAL) to *Ca*Las after being fed by the vector, *Diaphorina citri* (Asian citrus psyllid, or ACP). The results suggest the existence of a delayed defense reaction against the infective vector in VAL, while the attack by the healthy vector prompted immediate and substantial transcriptomic changes that led to the rapid erection of active defenses. Moreover, in the presence of *Ca*Las-infected psyllids, several downregulated differentially expressed genes (DEGs) were identified on the pathways, such as signaling, transcription factor, hormone, defense, and photosynthesis-related pathways at 1 day post-infestation (dpi). Surprisingly, a burst of DEGs (6,055) was detected at 5 dpi, including both upregulated and downregulated DEGs on the defense-related and secondary metabolic pathways, and severely downregulated DEGs on the photosynthesis-related pathways. Very interestingly, a significant number of those downregulated DEGs required ATP binding for the activation of phosphate as substrate; meanwhile, abundant highly upregulated DEGs were detected on the ATP biosynthetic and glycolytic pathways. These findings highlight the energy requirement of *Ca*Las virulence processes. The emerging picture is that *Ca*Las not only employs virulence strategies to subvert the host cell immunity, but the fast-replicating *Ca*Las also actively rewires host cellular metabolic pathways to obtain the necessary energy and molecular building blocks to support virulence and the replication process. Taken together, the very early response of citrus to the *Ca*Las, vectored by infective ACP, was evaluated for the first time, thus allowing the changes in gene expression relating to the primary mechanisms of susceptibility and host–pathogen interactions to be studied, and without the secondary effects caused by the development of complex whole plant symptoms.

## Introduction

Huanglongbing (HLB), also known as citrus greening, is a disease that affects all economically important citrus species, and some close citrus relatives (Folimonova et al., 2009; Ramadugu et al., 2016). This disease is associated with three species of Gram-negative α-proteobacteria, namely “*Candidatus* Liberibacter asiaticus (*Ca*Las),” “*Ca*. L. africanus,” and “*Ca*. L. americanus,” categorized according to their presumptive geographical origin and 16S rDNA molecular classification (Jagoueix et al., 1994; Li et al., 2006). They are non-culturable, phloem-limited, and characterized by transmission mediated by two species of citrus psyllid or by grafting (Bové, 2006; Wang and Trivedi, 2013). Decades of studies in HLB have expanded the knowledge of every aspect; however, due to its complexity until now, there have been no breakthroughs in HLB management (National Academies of Sciences, 2018).

In the plant-microbe battles, the ability to recognize and block microbial invasion is the key for hosts to launch defense responses and win the war against microbes. The defense mechanisms in plants triggered by pathogens have been well-studied and reviewed (Yu X. et al., 2017; Bendix and Lewis, 2018; Mermigka et al., 2020). In the case of HLB, *Ca*Las, the most prevalent species in major citrus-producing regions, is transmitted by its vector, Asian citrus psyllid (*D*. *citri*, ACP). Under its unculturable limitations, studies have revealed some of its possible pathogenic strategies from multiple genome sequences and their interaction with the hosts (Duan et al., 2009; Thapa et al., 2020). *Ca*Las is only able to metabolize a limited set of sugars, and probably uses exogenous molecules sourced from the phloem sap to generate energy (Duan et al., 2009). Indeed, 40 ATP-binding cassette (ABC) transporter proteins have been identified in the *Ca*Las genome, which is surprisingly higher than the average number of 15 in other intracellular bacteria with similar size (Davidson et al., 2008; Duan et al., 2009; Li et al., 2012). Among them, two ABC transporters have been reported in several studies to be associated with the virulence of the bacteria, which involved phosphate and zinc transport systems, respectively (Von Krueger et al., 1999; Garrido et al., 2003). Many of the candidate microbe-associated molecular patterns (MAMPs) have been identified in the *Ca*Las genome, and there is some evidence for the fact that *Ca*Las MAMPs are recognized in the host (Zou et al., 2012; Hao et al., 2013). Transcriptional analysis showed receptor-like kinases (RLKs), localized on the cell surface, were induced in *Ca*Las-infected citrus plants, suggesting that citrus host cells might recognize *Ca*Las MAMPs and initiate signaling cascades (Aritua et al., 2013; Mafra et al., 2013). Moreover, studies have computationally screened the pool of proteins with putative secretion signals in the *Ca*Las genome for potential effector candidates; yet, it is unknown what delivery system *Ca*Las uses (Pitino et al., 2016). Nevertheless, an effector CLIBASIA_05315 was revealed by a protein function study, which contributes to excessive cellular starch accumulation in *Nicotiana benthamiana*, a typical physiological disorder associated with *Ca*Las-infected citrus plants (Pitino et al., 2018). A further study characterized CLIBASIA_05315 and identified its target, a papain-like cysteine protease in citrus, which uncovered an interesting aspect of the virulence mechanism of HLB (Clark et al., 2018).

An important factor for the severity of the citrus HLB disaster is that there are no known HLB-resistant citrus species or varieties, or scion-rootstock combinations (Folimonova et al., 2009; Fan et al., 2013; Ramadugu et al., 2016). Several studies have demonstrated the complexity of pathogen, vector, and host interactions, and revealed that *Ca*Las infection primarily affects source-sink relationships, signaling pathways, and nutrient distribution, thereby creating environments favorable for colonization and proliferation within citrus plants (Martinelli et al., 2012; Zhao et al., 2013; National Academies of Sciences, 2018). Transcriptomic profiling of citrus–*Ca*Las interactions have been performed through microarray and high-throughput sequencing technologies, with different citrus tissues and pretreatments from both sensitive and tolerant citrus species (Albrecht and Bowman, 2008; Fan et al., 2012; Liao and Burns, 2012; Martinelli et al., 2012; Aritua et al., 2013; Xu et al., 2015; Yu Q. et al., 2017; Arce-Leal et al., 2020). Interestingly, on the one hand, even in the sensitive varieties, studies revealed that large numbers of defense-related genes are induced after *Ca*Las infection, which means that the citrus plants have the ability to launch the defense response, despite mostly losing the battle (Kim et al., 2009; Fan et al., 2012; Koh et al., 2012). On the other hand, more sensitive varieties have comparatively increased the expression levels of genes involved in callose deposition and cell-wall breakdown, but tolerant varieties exhibit increased expression levels of NBS-LRR, RLK, cell wall biosynthesis, and pathogenesis-related (PR) genes (Fan et al., 2012; Mafra et al., 2013; Wang et al., 2016). These findings could indicate that somehow the sensitive varieties are delayed in their defense responses to prevent pathogen spread, or the effector-triggered immunity is activated more rapidly in tolerant varieties. Therefore, it seems that the timing and the intensity of defense responses play a key role in this host–pathogen battle.

To understand the susceptibility of most citrus varieties, it is crucial to reveal the interaction between citrus and psyllid/*Ca*Las during the early phase of infection. However, most previous transcriptomic studies performed the inoculation through grafting with *Ca*Las-infected budwood or shoots, and the earliest sample time point was 5 weeks after grafting (Albrecht and Bowman, 2008, Fan et al., 2012), showing the limitations of this method of inoculation for studying the events that occur during the early infection stages. Recently, a study with detached citrus leaves showed that the *Ca*Las effector mRNA could be detected 6 h after ACP infestation (Shi et al., 2019). Therefore, in the present work, inoculation was performed through psyllid feeding on new flushes arising from *in vitro* cultured Valencia sweet orange (VAL) budwood, effectively avoiding environmental factors, and most importantly allowing transcript sampling at the very early infection phases, such as at 1 and 5 dpi, respectively.

## Methods

### Experimental Design

**Figure 1:**
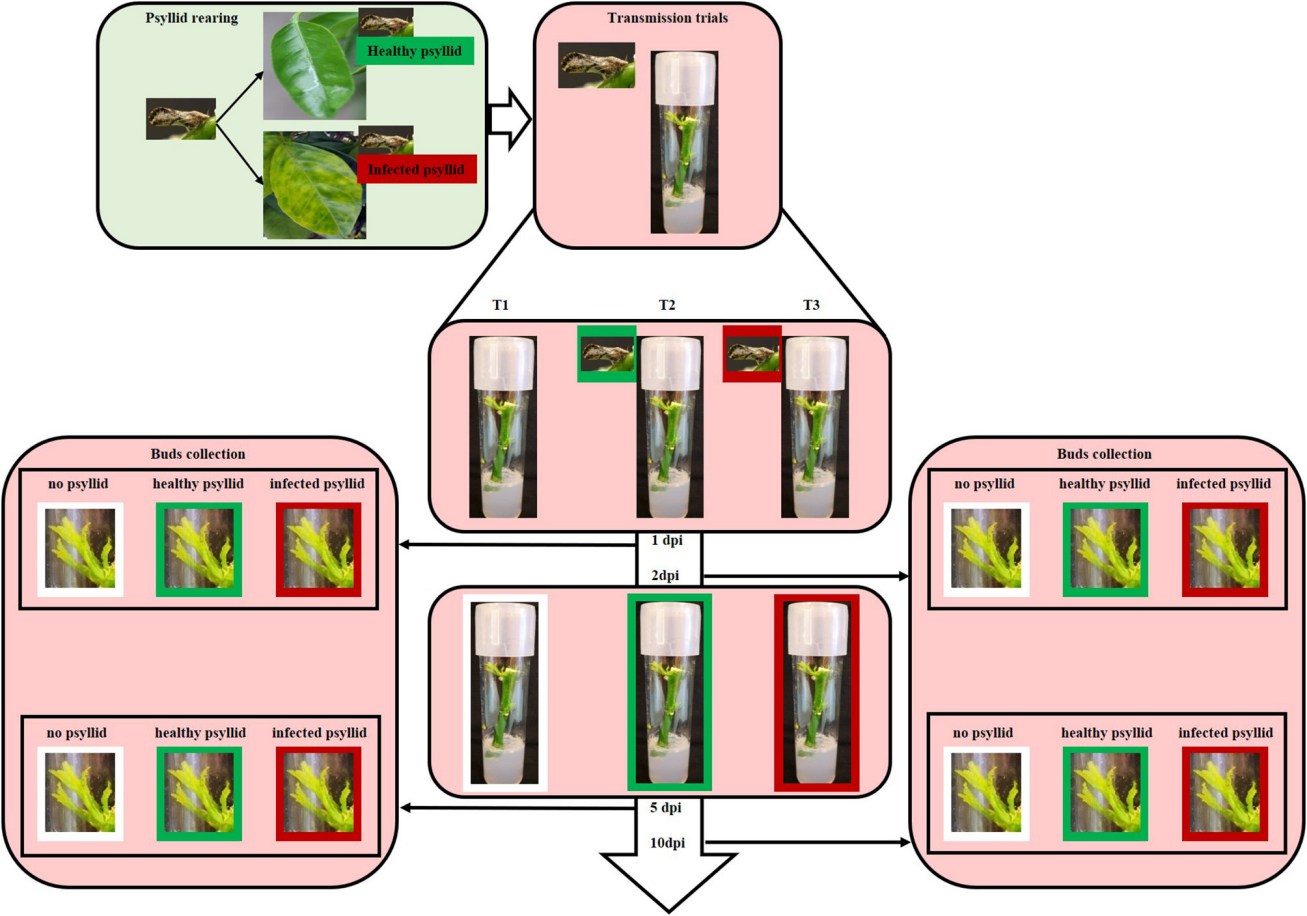
Diagram illustrating the rearing and infection of *Ca*Las and the transmission trials. Colonies of healthy psyllids were reared on HLB-free curry leaf plants in a growth room. *Ca*Las- infected psyllids were maintained on *Ca*Las-infected citrus trees in a growth room at the Citrus Research and Education Center of the University of Florida. For the transmission trials, five healthy or infected adult psyllids were put into each respective tissue culture tube when leaves of budwood sprouts began to unfold. Tissue was harvested from some of the plantlets at 1, 2, 5, and 10 days post-infection (dpi).

A completely randomized design was used in this study (Table 1, Figure 1). Healthy and clean budwood of VAL (*C. sinensis* L. Osbeck) were collected from greenhouse grown, certified pathogen-tested trees from the Florida Department of Agriculture and Consumer Services, Division of Plant Industry (FDACS-DPI) Bureau of Citrus Budwood Registration. The size of the budwood sticks was between 5 and 8 cm. These were surface-sterilized in 20% Clorox® regular bleach (3% sodium hypochlorite, v: v) for 5 min, followed by 70% ethanol for 10 s, and then washed three times with sterilized water for 10 s. After drying for a short time, they were transferred to test tubes measuring 25 × 100 mm containing Murashige and Skoog **(**MS) culture media and kept at 25 ± 1°C, 16:8 h photoperiod, and 60 μ mol^−2^ s^−1^ cool-white light. *Diaphorina citri* were introduced when leaves of budwood sprouts were unfolding (Figure 1). Colonies of healthy *D. citri* were reared on HLB-free curry leaf plants (*Bergera* [*Murraya*] *koenigii*) in a growth room maintained at 28 ± 1°C and 60 ± 5% relative humidity, and with a 16:8 h (L:D) photoperiod. *Ca*Las-infected psyllids were maintained on *Ca*Las-infected citrus trees in a growth room at the Citrus Research and Education Center of the University of Florida; these psyllids were shown to be 100% infected with *Ca*Las (Pandey et al., 2020). Five adult psyllids were released into each tube and allowed to feed on the tender, expanding shoots and leaves. Three feeding treatments were used for the experiment on the leaf sample collection: no psyllid (T1), healthy psyllid (T2), and *Ca*Las-infected psyllid (T3). Leaf samples were collected after psyllid feeding at 1 and 5 dpi. Three biological replicates were used for each treatment and time combination (except only two replicates were available for the 5 dpi with *Ca*Las-infected psyllids combination, because of excessive contamination growth *in vitro*).

**Table 1:** Experimental plan: cultivar, collection time, and experimental treatments.

	**Cultivar**	**Valencia sweet orange**			
	**Time point**	**1 dpi**	**2 dpi**	**5 dpi**	**10 dpi**
Treatment	No psyllid	No psyllid_1 dpi	No psyllid_2 dpi	No psyllid_5 dpi	No psyllid_10 dpi
	Healthy psyllid	Healthy psyllid_1 dpi	Healthy psyllid_2 dpi	Healthy psyllid_5 dpi	Healthy psyllid_10 dpi
	C*a*Las-infected psyllid	C*a*Las-infected psyllid_1 dpi	C*a*Las-infected psyllid_2 dpi	C*a*Las-infected psyllid_5 dpi	C*a*Las-infected psyllid_10 dpi

*CaLas, Candidatus Liberibacter asiaticus; Psyllid, Diaphorina citri, vector of CaLas; dpi, days post-infestation*.

### RNA Extraction, Complementary DNA Library Construction, and Sequencing

Total RNA was extracted as described previously (Yu Q. et al., 2017). In brief, TRIzol® Reagent (Invitrogen, Carlsbad, CA, United States) was used following the protocol by the manufacturer. Extracted RNA was further purified using the TURBO DNA-free™ kit to eliminate genomic DNA (Thermo Fisher Scientific Baltics UAB, Vilnius, Lithuania). RNA quality was assessed using NanoDrop One Spectrophotometer (Thermo Fisher Scientific, Waltham, MA, United States), gel electrophoresis, and Agilent 2100 Bioanalyzer (Aglient Technologies Inc., Waldbronn, Germany), respectively. The mRNAs were purified from total RNA using oligo (dT)-attached magnetic beads. Then first-strand of complementary DNA (cDNA) was generated using a random hexamer-primed reverse transcription, followed by a second-strand of cDNA synthesis. The synthesized cDNA was subjected to end-repair and then was 3'adenylated. Adapters were ligated to the ends of these 3'adenylated cDNA fragments, followed by PCR amplification and product purification using Ampure XP Beads (Agencourt Bioscience, Beverly, MA, United States). Agilent 2100 Bioanaylzer and ABI StepOnePlus Real-Time PCR System (Applied Biosystems, Foster City, CA, United States) were used in the quantification and qualification of those libraries. Seventeen cDNA libraries were constructed using an Illumina kit and sequenced on an Illumina HiSeq^TM^2100 platform employing paired-end (100 bp) technology at BGI Genomics Co., Ltd. (Shenzhen, China). The raw data of all RNA-seq samples were deposited in the NCBI Sequence Read Archive with the accession number SRP271181. The file of gene expression levels was deposited in the NCBI Gene Expression Omnibus (GEO) with the accession number, GSE154151.

### RNA-Seq Data Processing and Bioinformatics Analysis

To acquire valid sequencing data, the raw data were filtered using the SOAPnuke Toolkit software (v1.5.2). The sequencing reads containing low-quality, adaptor-polluted, or the high content of unknown bases were processed and removed. The clean reads were mapped to the *Citrus clementina* transcripts (https://phytozome-next.jgi.doe.gov/info/Cclementina_v1_0) using Bowtie2 (v2.2.5), and the gene expression levels were calculated with RNA-Seq by Expectation-Maximization (RSEM) tool (Li and Dewey, 2011). The gene expression level analysis of RNA-seq data was estimated by fragments per kilobase of transcript per million mapped reads (FPKM) (Trapnell et al., 2010). Data normalization and call of differentially expressed genes (DEGs) were implemented with the EBseq (v1.17.0)R (v3.2.0) package by setting the algorithms with |log2 ratio n| ≥ 1 and false discovery rate (FDR) ≤ 0.05, and by enabling independent filtering (Leng et al., 2013).

The annotation and analysis of DEGs were performed using the gene ontology sequencing (GOseq) (v1.26.0) R (v3.2.0) packages based on Wallenius' non-central hypergeometric distribution and the right-sided Fisher's exact test (Young et al., 2010). The Kyoto Encyclopedia of Genes and Genomes (KEGG) was used to interpret the utilities of biological systems and high-level functions of unigenes (Kanehisa et al., 2007). GO and KEGG enrichment analysis were conducted using a hypergeometric test with an FDR ≤ 0.01 adjusted *p*-value, to identify significantly enriched GO terms and the main biological pathways of DEGs with the entire transcriptome background. The DEG sequences were also assigned to several function groups of The Arabidopsis Information Resource (TAIR) database by Mercator sequence annotation tool (https://www.plabipd.de/portal/mercator-sequence-annotation) and by MapMan analysis (Thimm et al., 2004; Lohse et al., 2014). Log2 fold change was represented in MapMan as gene expression differences. The DEGs were analyzed by PageMan embedded in MapMan, and data were processed using Wilcoxon analysis with Fisher's exact test, setting a threshold of 1 (at least a 2-fold change). For the protein-protein network analysis, the DIAMOND/reference (v0.8.31) was used to map the DEGs with the STRING (v10) database to obtain the interaction between DEG-encoded proteins using homology with known proteins.

### Gene Expression Validation

To validate the results of DEGs identified by RNA-seq, 15 candidate modulated genes [8 from the ATP biosynthetic pathway, 6 from the glycolytic pathway (both pathways were shown to be significantly impacted), and 1 PR gene] were selected for quantitative real-time PCT (qRT-PCR) (Supplementary Material 1, Supplementary Table 1). Primers were designed based on cDNA sequences and 18S was used as an internal control. Samples collected at 2 and 10 dpi, respectively were used for qRT-PCR analysis, because of limited amounts of tissue available from 1 and 5 dpi. Though this is not ideal, we assumed that gene expression in samples collected at 1 and 2 dpi, and in samples collected at 5 and 10 dpi, respectively, should have similar trends. The geometric averaging method was used to normalize the qRT-PCR result, followed by the comparative Ct method (2^ΔΔCt^) (Vandesompele et al., 2002; Erickson et al., 2009). For each sample, the Log2 fold change was obtained from the ratio of the relative expression value of healthy vs. no psyllid control, and *Ca*Las-infected vs. no psyllid control. Log2 fold-changes of qRT-PCR were compared with the same gene from RNA-seq analysis (Supplementary Material 2, Supplementary Figure 1).

## Results

### Sequencing and Assembly of the VAL Transcriptome

The average total clean reads for each sample were 136.80 million (Supplementary Material 1, Supplementary Table 2). The average total mapping ratio to the reference transcripts achieved 61.24%. To search for important early *Ca*Las-response genes, the expression levels of the transcripts were calculated utilizing the FPKM approach (Supplementary Material 2, Supplementary Figure 2). We found that more genes had high expression (FPKM ≥10, avg. 10.571) and medium expression (FPKM1~10, avg. 9,325), and fewer genes exhibited low expression (FPKM ≤ 1, avg. 5,839) in all the 17 libraries.

### Identification of DEGs

**Figure 2:**
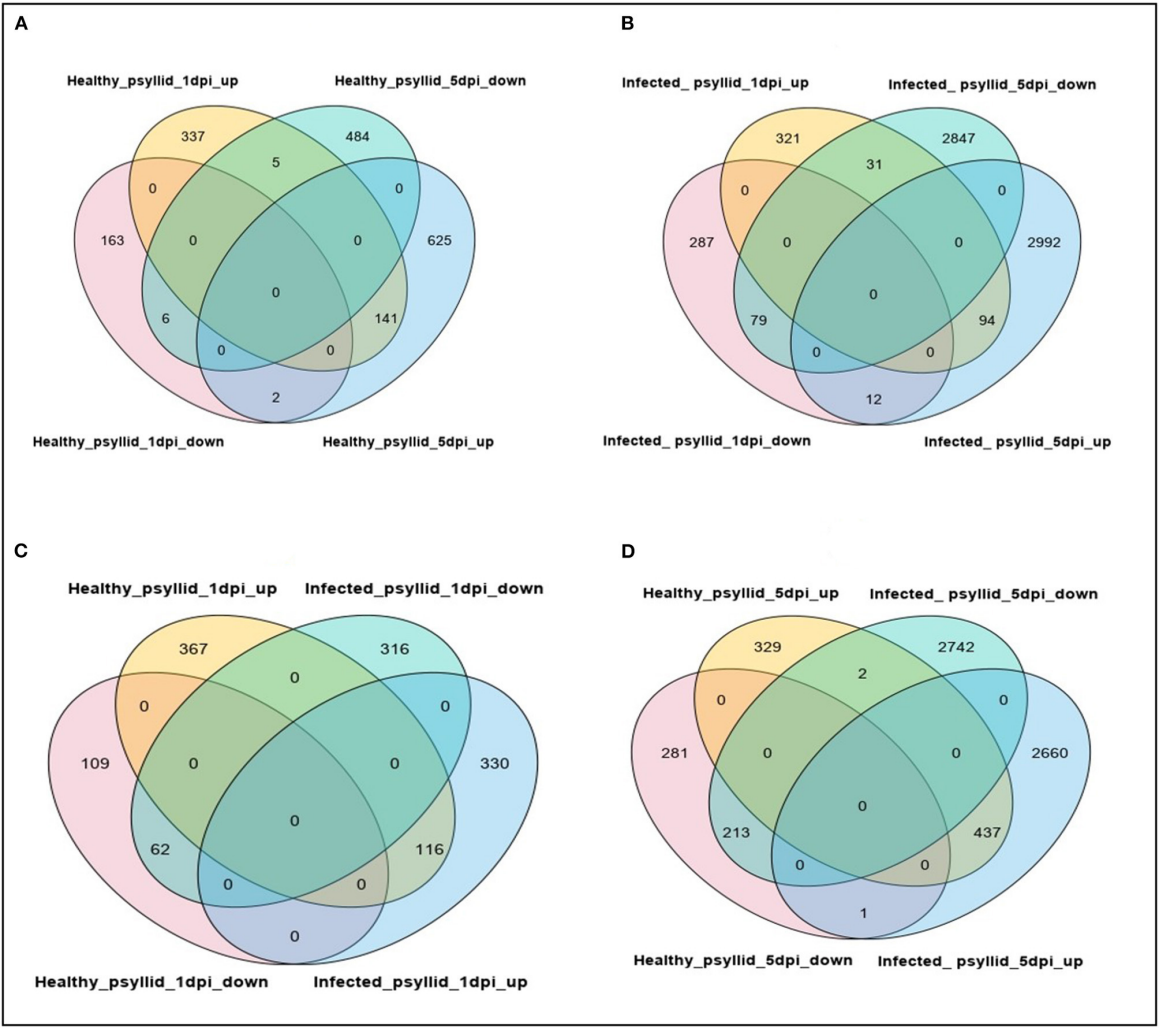
Venn diagrams showing numbers of upregulated and downregulated DEGs after healthy and *Ca*Las-infected psyllid attacks in Valencia sweet orange, at different time points post-infestation. **(A)** Healthy_psyllid_at 1 dpi vs. healthy_psyllid at 5 dpi; **(B)** Infected_psyllid_at 1 dpi vs. Infected_psyllid_at 5 dpi; **(C)** Healthy_psyllid_at 1 dpi vs. infected_psyllid_at dpi; **(D)** Healthy_psyllid_at 5 dpi vs. Infected_psyllid_at 5 dpi.

Pairwise comparisons between no psyllid_1 dpi and healthy_psyllid_1 dpi, no psyllid_1 dpi and *Ca*Las-infected_psyllid_1 dpi, no psyllid_5 dpi and healthy_psyllid_5 dpi, no psyllid_5 dpi, and *Ca*Las-infected_psyllid_5 dpi resulted in four sets of regulated genes, respectively. Those genes with a fold change of at least 2 (|log2FC| ≥ 1), and with a posterior probability of equivalent expression (PPEE) < 0.05 were considered DEGs (Supplementary Material 1, Supplementary Tables 3–6). In Table 2, under the healthy psyllid infestation, the numbers of DEGs were found to be 654 and 1,263 at 1 and 5 dpi, respectively; the numbers of DEGs were found to be 824 and 6,055 at 1 and 5 dpi under *Ca*Las-infected psyllid infestation, respectively. The result indicated that there were more DEGs in response to *Ca*Las-infected psyllid infestation than to the healthy psyllid infestation at both time points. Moreover, the analysis of the common DEGs between the two different treatments and two time points was conducted, and the results indicated 92% (141) of the common DEGs (154) under the healthy psyllid treatment between 1 and 5 dpi were co-upregulated, while under the *Ca*Las-infected psyllid infestation between 1 and 5 dpi, 44% (94) of the common DEGs (216) were co-upregulated and 36% (79) were co-downregulated (Figure 2).

**Table 2:** Number of DEGs and enriched GO terms of the major biological process classes in the comparisons “No psyllid vs. Healthy psyllid” and “No psyllid vs. *Ca*Las-infected psyllid” in Valencia orange.

	**No psyllid vs. Healthy psyllid**		**No psyllid vs**. ***Ca*****Las-infected psyllid**	
	**1 dpi**	**5 dpi**	**1 dpi**	**5 dpi**
DEGs total	654	1,263	824	6,055
DEGs upregulated	483	768	446	3,098
DEGs downregulated	171	495	378	2,957
Enriched GO terms	32	35	34	38

**Figure 3:**
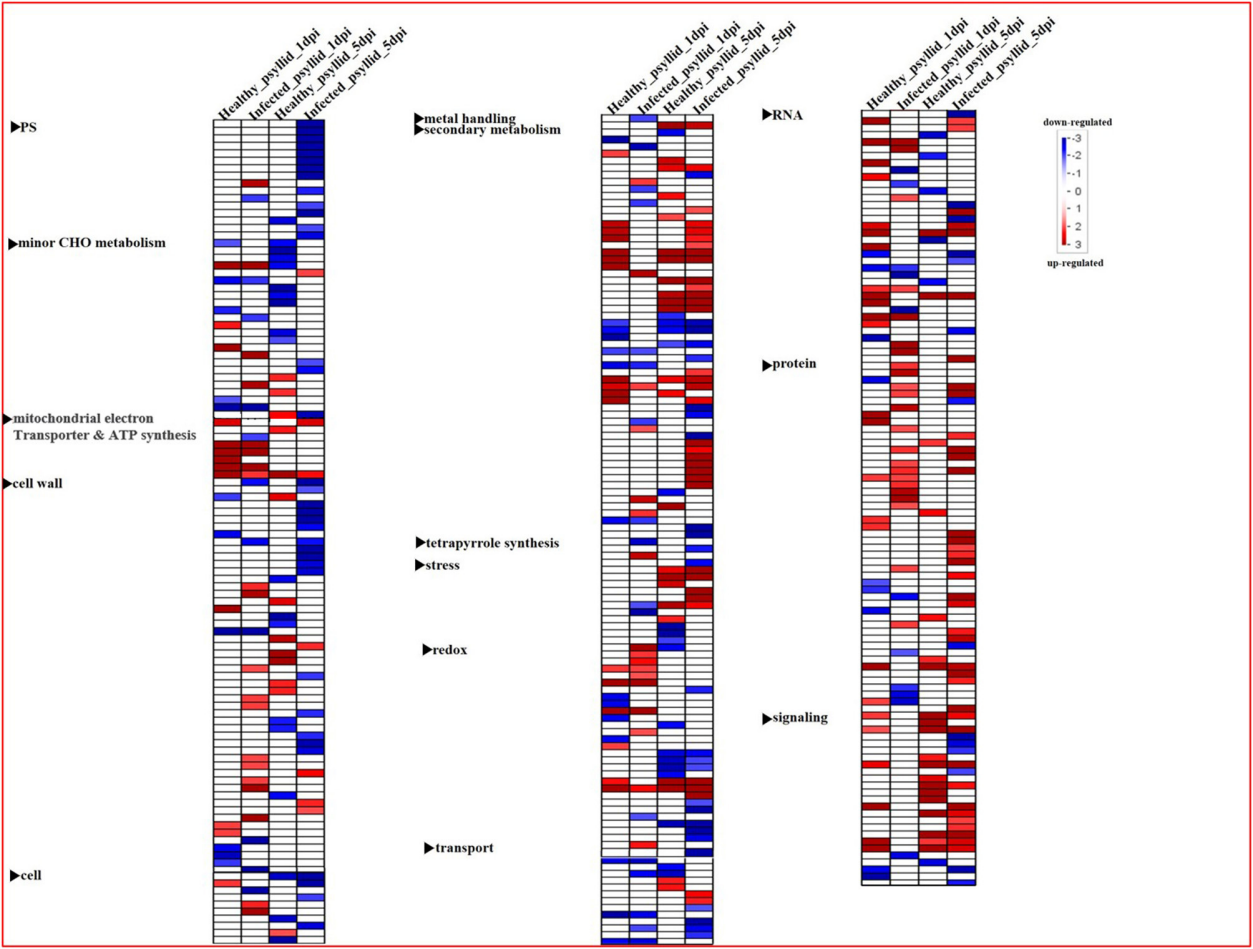
Overview of the significantly affected functional categories in Valencia sweet orange in healthy and *Ca*Las-infected psyllid treatments at 1 and 5 dpi. Changes in transcript levels are presented as log2 fold changes. The data were subjected to the Wilcoxon test in PageMan, and the results are displayed in false-color code. Bins colored in red are significantly upregulated, whereas bins colored in blue are significantly downregulated. PS, photosynthesis.

To gain a holistic understanding of the functional roles of the DEGs, the Wilcoxon rank-sum test analysis was conducted by PageMan integrated into the MapMan software, and different sets of enriched bins were identified, modulated by healthy and *Ca*Las-infected psyllid infestation at 1 and 5 dpi, respectively (Figure 3). In VAL under the healthy psyllid infestation, a large number of bins with upregulated and downregulated genes related to signaling and secondary metabolism were enriched at 5 dpi. In VAL under *Ca*Las-infected psyllid infestation, only one bin with downregulated genes in signaling was enriched at 1 dpi, while a large number of bins with upregulated genes in protein metabolism, and all functional categories, were enriched at 5 dpi; these included most bins with downregulated genes related to photosynthesis (PS) and cell wall metabolism and many bins with upregulated genes related to secondary and protein metabolism. The above analysis indicated that treatments and time points both played important roles in defining the overall transcriptomic dynamics.

### Gene Ontology Enrichment and KEGG Enrichment Analysis of DEGs

Because of the absence of the appropriate bins for many species in MapMan, GO enrichment and KEGG pathway enrichment analyses were performed using the DEGs, as complementary sources of information. The DEGs were classified by GO terms into three functional categories: biological process, molecular function, and cellular component (Supplementary Material 2, Supplementary Figure 3). Under the healthy psyllid infestation, the GO enrichment analysis yielded 32 and 35 enriched GO terms, at 1 and 5 dpi, respectively, with 32 common GO terms (Supplementary Material 2, Supplementary Figures 3A,C), while an increased number of enriched GO terms under the *Ca*Las-infected psyllid infestation was found to be 34 and 38 at 1 and 5 dpi, respectively. Thirty-four GO terms were common between the two time points (Supplementary Material 2, Supplementary Figures 3B,D). Notably, in the most enriched GO terms, more DEGs were found downregulated in response to the *Ca*Las-infected psyllid infestation than to healthy psyllid at both time points. Similarly, all DEGs were further classified by KEGG pathways enrichment analysis (Supplementary Material 2, Supplementary Figure 4). These pathways included plant hormone signal transduction, biosynthesis of secondary metabolites, glutathione metabolism, protein processing in the endoplasmic reticulum, and metabolic pathways, among others. At 1 dpi, DEGs in plant hormone signal transduction pathways were significantly downregulated under the *Ca*Las-infected psyllid infestation, and all DEGs were upregulated on the protein processing in the endoplasmic reticulum pathway, while most of DEGs on both glutathione metabolism and plant hormone signal transduction pathways were upregulated under the healthy psyllid attacks. At 5 dpi, the metabolic pathway was the major pathway that was significantly enriched under healthy and *Ca*Las-infected psyllid infestation; however, more DEGs were upregulated under healthy psyllid infestation, while more were downregulated under *Ca*Las-infected psyllid infestation. The result of GO and KEGG analysis showed a higher number of downregulated DEGs under the *Ca*Las-infected psyllid infestation than the healthy psyllid infestation at both time points, indicating that the *Ca*Las may have an overwhelming effect on the genes of those significant downregulated pathways at the very early stage. Moreover, several affected metabolic pathways were involved in the plant reaction to biotic stress (Supplementary Material 2, Supplementary Figure 5). The categories of genes modulated after healthy and *Ca*Las-infected psyllid infestation in VAL are described in detail in the following sections.

### Signaling Pathways

In plants, several signal transduction pathways, largely governed by protein kinases and Ca^2+^ are involved in response to adverse environmental stimuli (Clark et al., 2001). In the present study, these pathways were significantly modulated in the different treatments (Supplementary Material 2, Supplementary Figure 5; Supplementary Material 1, Supplementary Tables 7, 8).

**Figure 4:**
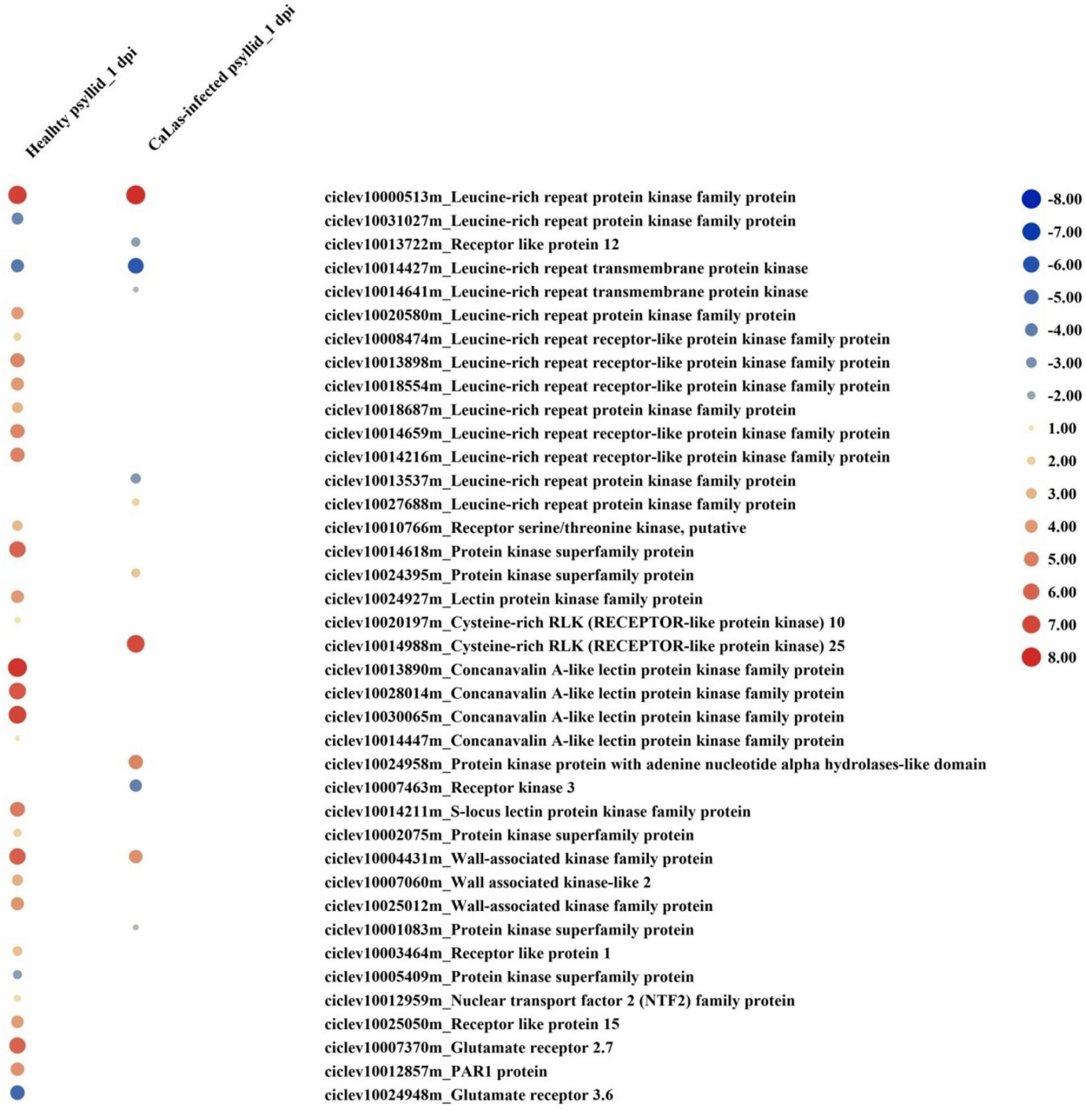
The red-blue heatmap of modulated DEGs involved in “signaling receptor” pathways. Each DEG is depicted by a color signal where red signifies upregulation and blue signifies downregulation. The size of each spot indicates the level of expression.

At 1 dpi, the expression of many gene codings for different groups of protein kinases, mainly receptor-like kinases (RLKs) and receptor-like proteins (RLPs), was modulated in response to healthy psyllid infestation (Figure 4). In total, 30 DEGs encoding receptor proteins were identified, and most of them were upregulated including some *RLK* and *leucine-rich repeats* (*LRRs*) family members, which had been reported in plant-microbe interactions and stress response (Shiu and Bleecker, 2001). The protein kinase genes were also present among DEGs in VAL under *Ca*Las-infected psyllid infestation, but only 12 genes were present among coding receptor proteins. Most of the *LRR* genes (6 out of 8) were downregulated (Figure 4). At 5 dpi, 90 genes of VAL response to healthy psyllid infestation were characterized by a general upregulation of the expression of *RLKs* belonging to different families; however, the *Ca*Las-infected psyllid infestation resulted in 260 DEGs. Specifically, 73 of 118 *LRR* genes were downregulated. It is very interesting to find that at 1 dpi, two *GLRs* (*glutamate receptors*) (upregulated *GLR2.7* and downregulated *GLR3.6*) were significantly modulated in VAL under healthy psyllid infestation, while no *GRLs* was significantly regulated in VAL under *Ca*Las-infected psyllid infestation (Figure 4). At 5 dpi, several *GLRs* were upregulated in response to healthy and *Ca*Las-infected psyllid infestation with different regulation patterns.

**Figure 5:**
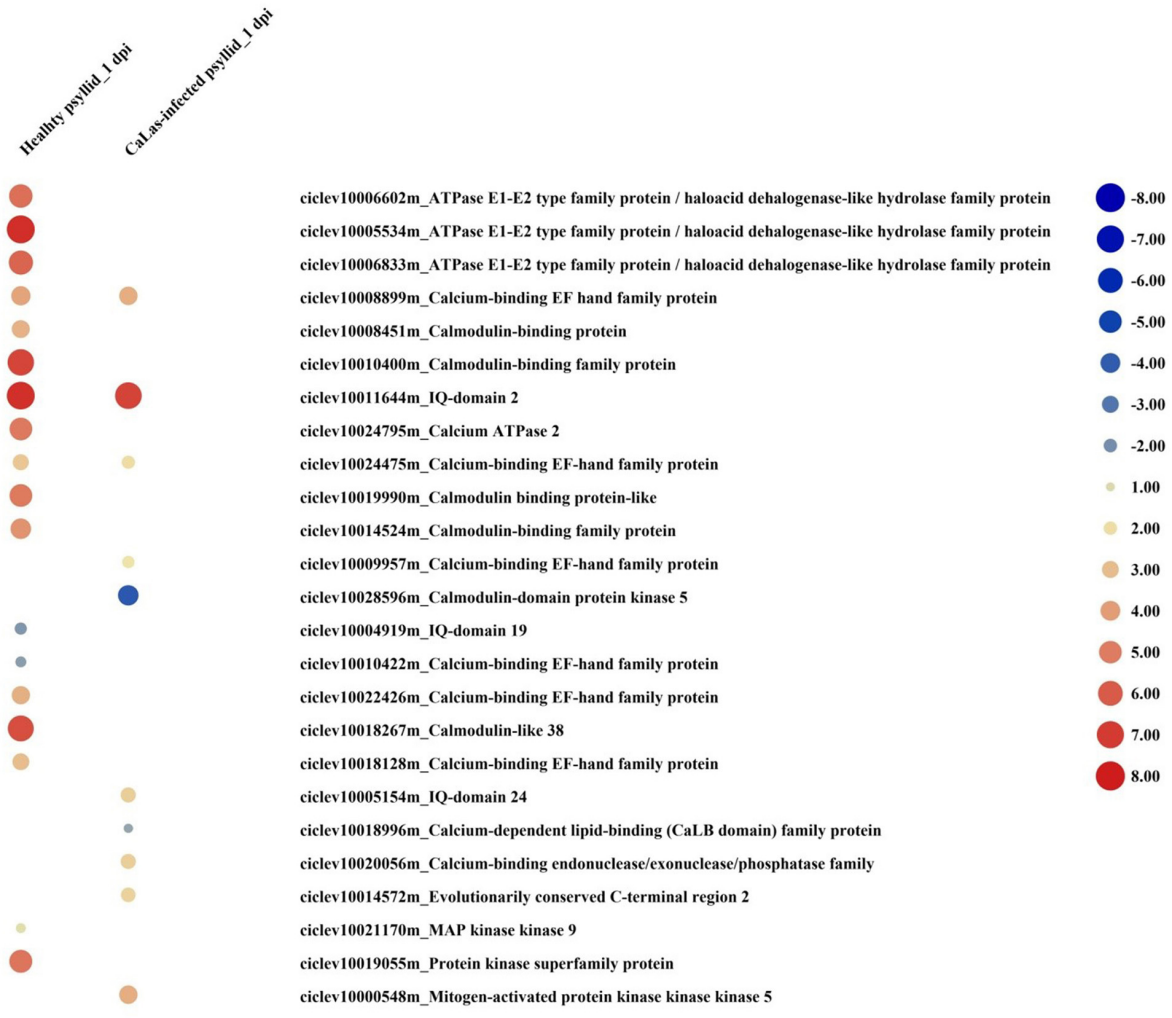
The red-blue heatmap of modulated DEGs involved in “signaling calcium” and “signaling MAP kinases” pathways. Each DEG is depicted by a color signal where red signifies upregulation and blue signifies downregulation. The size of each spot indicates the level of expression.

At 1 dpi, 14 out of 16 upregulated DEGs were identified as Ca^2+^-mediated signaling-associated genes in response to healthy psyllid infestation. There were seven upregulated and two downregulated Ca^2+^-mediated signaling DEGs detected in response to *Ca*Las-infected psyllid infestation (Figure 5). Notably, one of the significantly downregulated DEGs, *calmodulin-domain protein kinase CDPK isoform 5* (*CPK5*) (ciclev10028596m), has been shown to be a central hub in the local and distal immune signaling (Seybold et al., 2019). At 5 dpi, 21 out of 26 DEGs involved in calcium signaling were observed under the healthy psyllid infestation, while 47 out of 72 upregulated DEGs were found under the *Ca*Las-infected psyllid infestation. *Calmodulin-like 38* (*CML38*) was significantly accumulated in VAL under healthy psyllid attacks at 1 dpi, and several *CPKs* (*CPK5, CPK6, CPK9*, and *CPK16*) were significantly upregulated in VAL under *Ca*Las-infected psyllid infestation at 5 dpi. Moreover, some mitogen-activated protein (MAP) kinases were modulated under both treatments at two time points (Figure 5). Notably, a member of the MAP Kinase Kinase family (ciclev10021170m), which has been shown to be independently involved in ethylene biosynthesis, was upregulated in VAL under healthy psyllid infestation at 1 dpi.

### Hormone-Related Pathways

**Figure 6:**
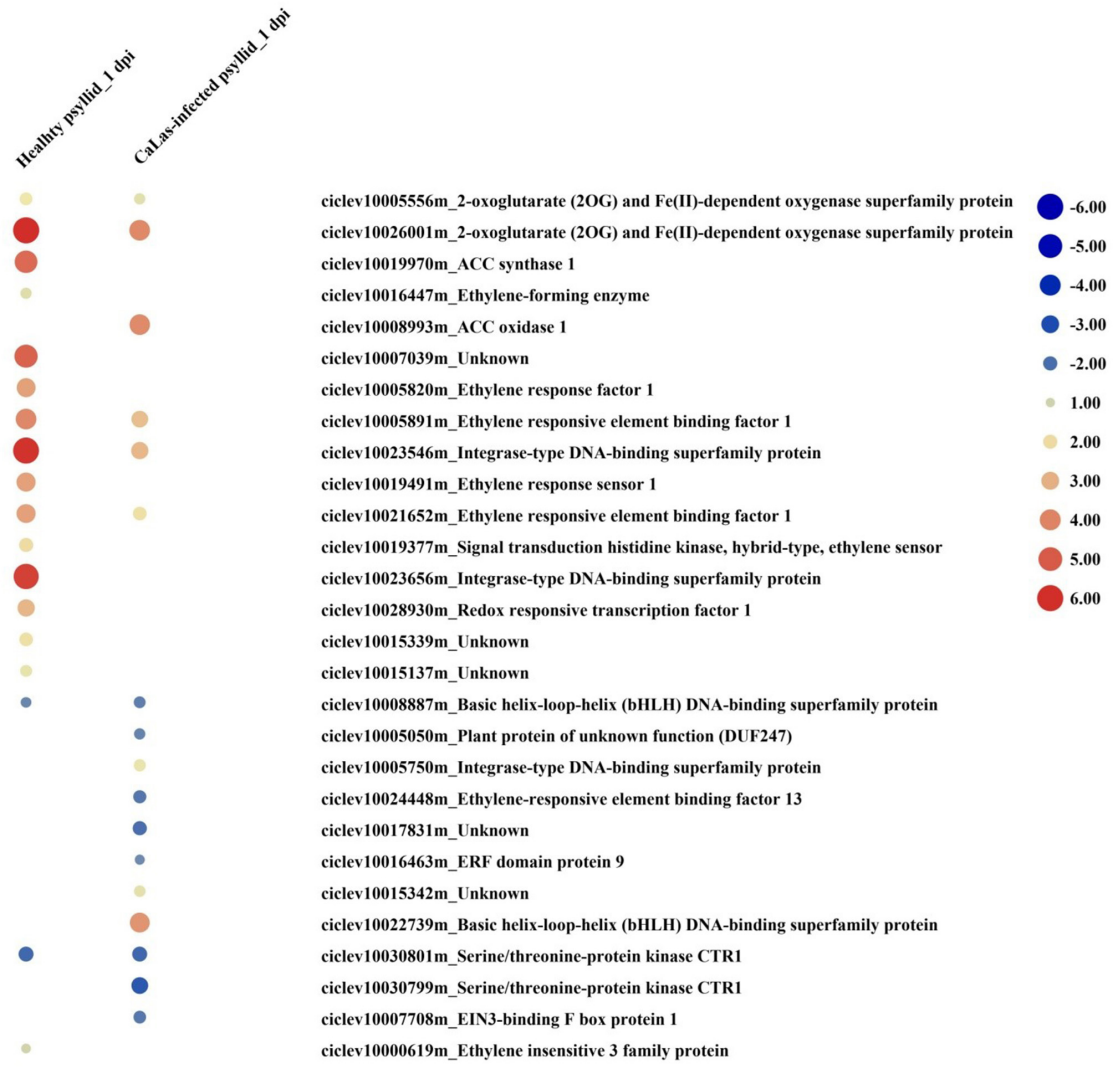
The red-blue heatmap of modulated DEGs involved in ethylene signaling pathways. Each DEG is depicted by a color signal where red signifies upregulation and blue signifies downregulation. The size of each spot indicates the level of expression.

Hormone signaling pathways strongly affect the timing and intensity of disease responses in plants. DEGs in signaling pathways, such as ethylene (ET), abscisic acid (ABA), auxin (AUX), brassinosteroid (BR), jasmonic acid (JA), and salicylic acid (SA) were identified under both treatments at both time points (Supplementary Material 2, Supplementary Figure 5; Supplementary Material 1, Supplementary Tables 9, 10). At 1 dpi, DEGs involved in ABA, auxin, BR, and ethylene synthesis, and signal transduction pathways were detected. Under the healthy psyllid infestation, the expression of genes of the ethylene synthesis and transduction pathways were strongly upregulated (Figure 6), including one *1-aminocyclopropane-1-carboxylate synthase* (*ACS*), one *1-aminocyclopropane-1-carboxylate oxidase 1*(*ACO*), two ethylene receptor sensors (*ERSs*), seven ethylene response factors (*ERFs*), and one downregulated basic helix-loop-helix (*bHLH*), while under the *Ca*Las-infected psyllid infestation, one *ACO*, four *ERFs*, and one *bHLH* were upregulated, the other two *ERFs* and one *bHLH* were downregulated. Moreover, according to the GO annotation (Supplementary Material 1, Supplementary Table 10), under the healthy psyllid infestation, an *ethylene insensitive3* (*EIN3*) (ciclev10000619m) and *EIN3-binding F box* (ciclev10007739m) were upregulated at 1 and 5 dpi, respectively; under *Ca*Las-infected psyllid infestation, an *EIN3-binding F box* (ciclev10007708m) was downregulated at 1 dpi, while two *EIN3* (ciclev10000606m, ciclev10014617m), and an *EIN3-binding F box* (ciclev10007739m) were upregulated at 5 dpi. The EIN3 transcription factor is the key regulator of ethylene signaling that sustains a variety of plant responses to ethylene.

### Transcription Factors

Many gene encoding transcription factors, including those belonging to the *WRKY*, myeloblastosis (*MYB*)*, apetala2* (*AP2*)*/ERF*, and *DNA binding with OneFinger* (*DOF*) families, were differentially expressed (Supplementary Figure 5; Supplementary Material 1, Supplementary Table 11). At 1 dpi, 20 out of 21 DEGs encoding *WRKY, MYB, AP2/ERF*, and *basic-leucine zipper* (*bZIP*) members were strongly upregulated under healthy psyllid infestation, while 9 of 17 genes on those pathways were downregulated under *Ca*Las-infected psyllid infestation. At 5 dpi, DEGs encoding *WRKY* and *MYB* members were strongly upregulated under infestation by healthy psyllid, while DEGs encoding *WRKY, MYB, AP2/ERF*, and *DOF* families were found under *Ca*Las-infected psyllid infestation. Notably, a *RelA-SpoT homolog* (*RSH*) (ciclev10019103m) was significantly downregulated in VAL after *Ca*Las-infected psyllid infestation at 1 dpi.

### Antioxidant Mechanisms

In plants, several enzymatic and non-enzymatic antioxidant mechanisms could be activated as protection against invading pathogens (Mendoza, 2011). DEGs involved in the control of the cellular redox state were modulated in response to healthy and *Ca*Las-infected psyllid feeding at both time points (Supplementary Figure 5; Supplementary Material 1, Supplementary Table 12). At 1 dpi, 23 and 18 upregulated DEGs were observed to respond to healthy and *Ca*Las-infected psyllid infestation, respectively; mainly among these, the non-enzymatic antioxidants which performed a detoxifying function on reactive oxygen species (ROS), such as ascorbate peroxidase (APX) and glutathione S-transferase (GST), together with those enzymatic antioxidants, such as those coded for superoxide dismutase (SOD) and peroxidase (POD), were present. At 5 dpi, the response trend was consistent as 1 dpi under healthy psyllid infestation, while the response burst under *Ca*Las-infected psyllid infestation.

### Defense Proteins

At 1 dpi, a few DEGs encoding various defense-related proteins, which can possess anti-insect and antimicrobial activities, were found in samples under both treatments (Supplementary Material 2, Supplementary Figure 5; Supplementary Material 1, Supplementary Table 13). Under the healthy psyllid infestation, seven disease resistance protein genes (*TIR-NBS-LRR* class) out of eight were significantly upregulated, and six of them involved in signal transduction. However, under the *Ca*Las-infected psyllid infestation, only three disease resistance protein genes out of nine were upregulated; among those six downregulated DEGs, four of them were involved in signal transduction. At 5 dpi, 19 upregulated defense DEGs were detected under healthy psyllid infestation, while there was a burst of DEGs under *Ca*Las-infected psyllid infestation, and most of those were repressed.

### Cell Wall Metabolism

At 1 dpi, cell wall metabolism was modulated differently in VAL under both treatments (Supplementary Material 2, Supplementary Figure 5; Supplementary Material 1, Supplementary Table 14); under the healthy psyllid infestation, two DEGs encoding xyloglucan endotransglycosylase-related protein (XTR6) and one DEG encoding pectin acetylesterase (PAE) family protein were significantly upregulated. Previous studies indicated that PAE functions as an important structural regulator in plants by modulating the precise status of pectin acetylation to affect the remodeling and physiochemical properties of the cell wall polysaccharides thereby affecting the cell extensibility (Gou et al., 2012). However, under the *Ca*Las-infected psyllid infestation, two DEGs encoding xyloglucan endotransglucosylase/hydrolase (XTH5, XTH32), one DEG encoding glycosyl transferase, four DEGs encoding pectin lyase-like superfamily protein, two DEGs encoding cellulose synthase, and one DEG encoding expansin-like were downregulated. All these DEGs have important roles in plant cell wall modification and formation. At 5 dpi, several genes involved in the hydrolysis of polysaccharides, like XTH, and pectin methylesterase and pectin acetylesterase were induced in VAL under healthy psyllid infestation, but again there was a burst of DEGs under *Ca*Las-infected psyllid infestation, and most of those were repressed.

### Secondary Metabolism

Several bins involved in the secondary metabolism were enriched (Figure 3; Supplementary Material 1, Supplementary Table 15). Generally, the modulation of the DEGs had similar upregulated trends under healthy psyllid infestation. Specifically, DEGs in phenolic metabolism were significantly upregulated, including *cinnamic acid 4-hydroxylase* (*C4H*), *4-coumaroyl-CoA synthase* (*4CL*), and *caffeic acid 3-O-methyltransferase* (*CAOMT*). Under the *Ca*Las-infected psyllid infestation, two *caffeoyl-CoA O-methyltransferases* (*CCoAOMT*) on the phenylpropanoid pathway were significantly downregulated at 1 dpi, and out of 60 DEGs, 31 were found downregulated at 5 dpi. There were several downregulated DEGs on the non-mevalonate pathway (MEP), including *1-deoxy-D-xylulose-5-phosphate synthase* (*DXS*) and *1-deoxy-d-xylulose 5-phosphate reductoisomerase* (*DXR*), which catalyze the first committed step of the 2-C-methyl-d-erythritol 4-phosphate pathway for isoprenoid biosynthesis, two *4-hydroxy-3-methyl but-2-enyl diphosphate reductase* (*HDR*), and one *3-hydroxy-3-methylglutaryl-coenzyme A reductase 2* (*HMG-CoA reductase 2*), which is involved in mevalonate biosynthesis and performs the first committed step in isoprenoid biosynthesis. Moreover, on the phenylpropanoids biosynthesis pathway, the *phenylalanine ammonia-lyase* (*PAL*), *4CL, hydroxycinnamoyl-CoA shikimate/quinate hydroxycinnamoyl transferase* (*HCT*), *CCoAOMT*, and *CAOMT* were downregulated. Notably, ten DEGs involved in carotenoid biosynthesis and three DEGs involved in terpenoid biosynthesis were downregulated.

### Primary Metabolism

Under the healthy psyllid attacks, eight genes on the primary metabolism pathways were significantly modulated at 1 dpi, particularly, with three upregulated DEGs related to PS and nucleotide metabolism pathways and three downregulated DEGs related to carbohydrate metabolism pathways (Supplementary Material 1, Supplementary Table 16). Thirty-seven genes were regulated at 5 dpi, particularly, with three downregulated DEGs coded for RubisCO involved in chloroplast precursor, three upregulated DEGs coded for adenylate kinase family protein involved in ATP/GTP biosynthesis, 13 downregulated DEGs involved in carbohydrate metabolism, and three downregulated DEGs involved in the fatty acid pathway. Notably, one DEG involved in callose synthesis (ciclev10030476m) was upregulated. These modulated DEGs indicated that the primary event in carbon dioxide fixation was downregulated, but the energy metabolism was upregulated. Under *Ca*Las-infected psyllid infestation, 20 genes were significantly modulated with 10 upregulated and 10 downregulated at 1 dpi. Notably, one upregulated DEG (ciclev10007488m) was involved in sucrose synthesis, one downregulated DEG (ciclev10030780m) was involved in starch degradation, and one downregulated DEG (ciclev10030481m) was involved in callose synthesis. The expression of many genes encoding the components of the photosystems, nucleotide metabolism, carbohydrate metabolism, lipid metabolism, and tetrapyrrole synthesis was significantly modulated at 5 dpi. Interestingly, most of the genes involved in nucleotide metabolism were upregulated; however, almost all the other genes involved in photosystems, lipid metabolism, and tetrapyrrole synthesis were significantly downregulated. Eight out of ten DEGs involved in starch synthesis were downregulated, including an *ADP-glucose synthase* which catalyzes the first, rate-limiting step in starch biosynthesis, one alpha-amylase, and four glycosyl hydrolase involved in starch degradation were upregulated. Notably, two *glucan synthase-like 7* (*GSL07*) were downregulated, while one *GSL03* was upregulated. The expression patterns of DEGs indicated that PS was severely downregulated; however, the energy metabolism was actively induced.

### Differentially Expressed Genes Requiring ATP/GDP/GTP and Phosphate for Function

**Figure 7:**
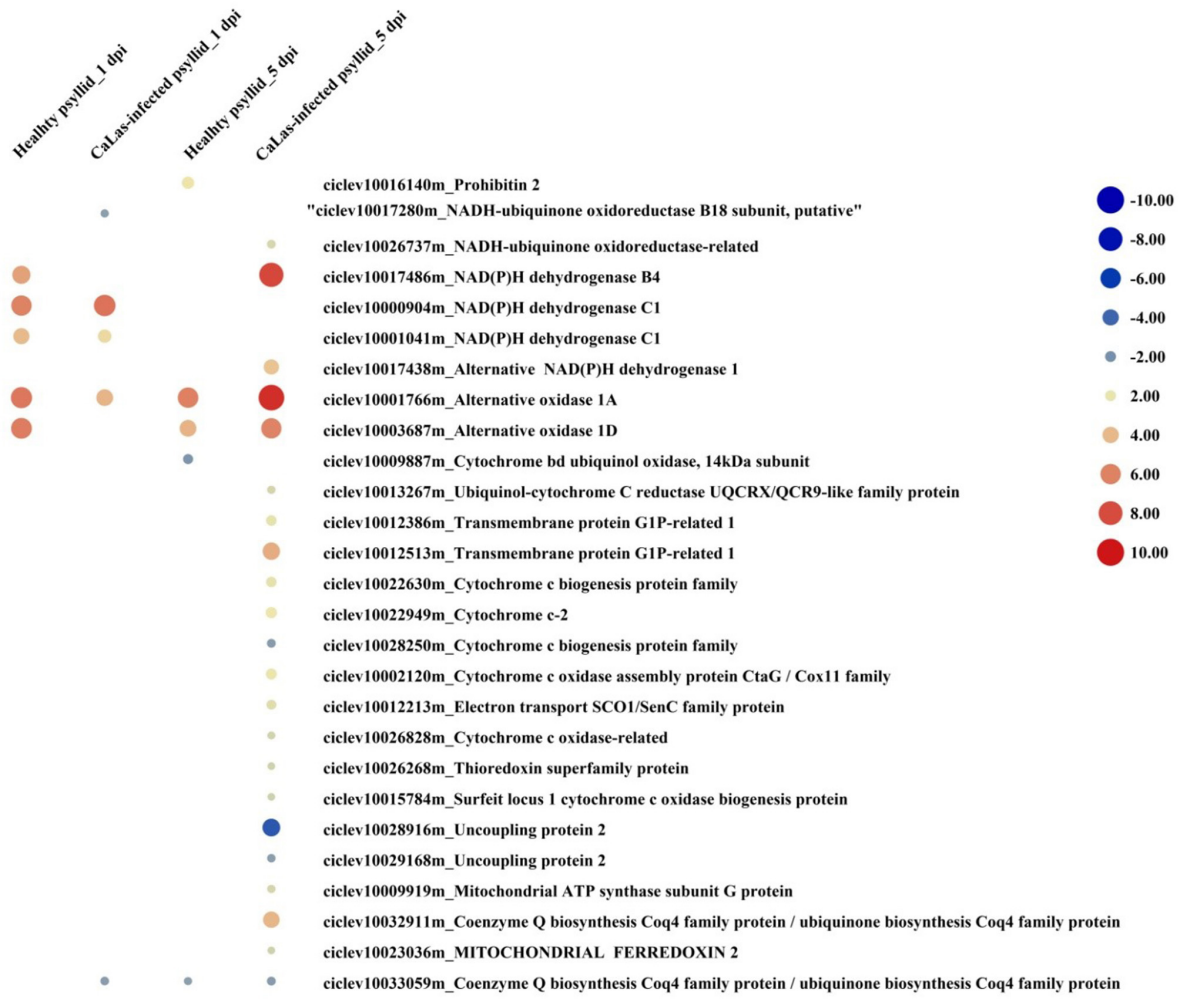
The red-blue heatmap of modulated DEGs involved in ATP biosynthesis pathways. Each DEG is depicted by a color signal where red signifies upregulation and blue signifies downregulation. The size of each spot indicates the level of expression.

**Figure 8:**
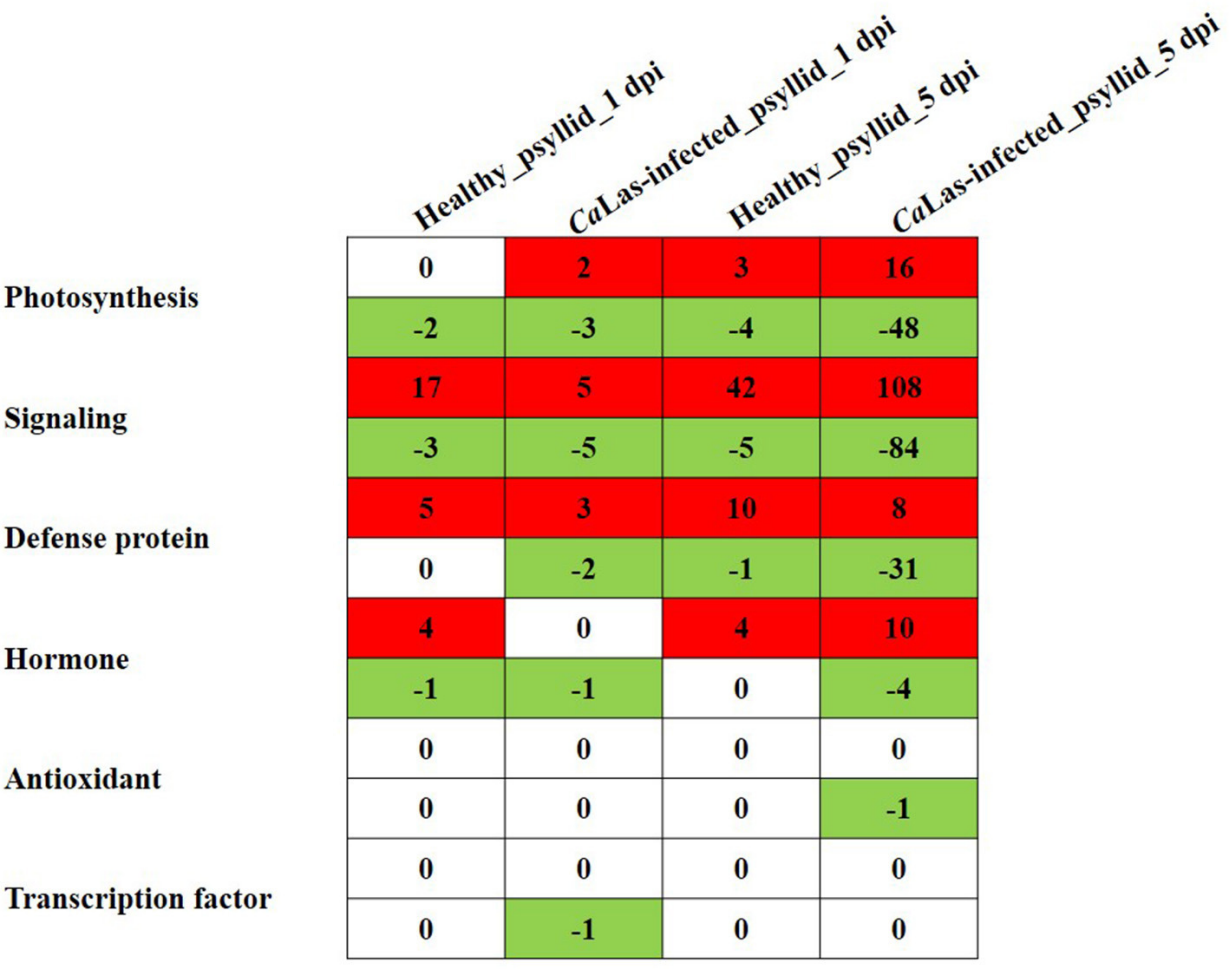
The number of DEGs involved in photosynthesis, signaling, hormone, defense protein, transcription factor, and antioxidant pathways detected in VAL at 1 and 5 days post healthy psyllid and *Ca*Las-infected psyllid infestation, which require ATP/GDP/GTP and phosphate for function. Cells colored in red represent upregulated DEGs and cells colored in green represent downregulated DEGs.

According to the above analysis, the defense response (the signaling and hormone pathways) of VAL to the *Ca*Las-infected psyllid infestation was suppressed at 1 dpi; however, it was strongly activated at 5 dpi. But why does VAL then lose the battle with *Ca*Las? We noticed significantly upregulated ATP biosynthesis pathways in VAL under *Ca*Las-infected psyllid infestation at 5 dpi (Figure 7; Supplementary Material 1, Supplementary Table 17), which indicated that plentiful ATPs were produced. Interestingly, the number of DEGs requiring ATP/GDP/GTP and phosphate for function under *Ca*Las-infected psyllid infestation was significantly higher than under healthy psyllid infestation (Figure 8; Supplementary Material 1, Supplementary Table 18). According to the MapMan annotation, among the DEGs involved in response to biotic stress, 369 of them were identified as requiring ATP/GDP/GTP and phosphate for functions, including 58 DEGs requiring phosphate which were mainly on the PS pathways, and 332 requiring ATP/GDP/GTP which were mainly on the pathways, such as signaling, defense protein, and hormone-related pathways. Moreover, the result also showed that the ATP/GDP/GTP/phosphate requiring DEGs in VAL under the *Ca*Las-infected psyllid infestation was repressed at 1 dpi, regardless of however strongly they were modulated up and down at 5 dpi. This finding indicated that the DEGs involved in energy metabolism and the DEGs requiring energy for function were significantly affected by *Ca*Las-infected psyllid infestation.

## Discussion

In this study, RNA-seq was used to compare the very early transcriptional changes occurring in VAL during the interaction between the pathogen *Ca*Las, its vector psyllid, and the host citrus. The study used ACP infestation with *in vitro*-cultured budwood for inoculation, aiming to mimic the natural event of infection in controlled conditions. The transcriptomic analysis showed that the plant response to pathogen invasion at 1 dpi was repressed in responses, such as the inactive signaling, ET, defense protein, and transcription factor-mediated response. However, there was a burst of gene modulation at 5 dpi, severely downregulated PS pathways, upregulated ATP, ET, and transcription factor synthesis pathways, and the combined upregulation and downregulation in the signaling, defense protein, cell wall, and secondary metabolism pathways. In this study, the very early response of citrus to the *Ca*Las vectored by infective ACP was evaluated for the first time, thus allowing for the changes in gene expression to be studied relating to the primary mechanisms of susceptibility and host-pathogen interactions, and without the secondary effects caused by the development of complex whole plant symptoms. In addition, the already-present repression mechanisms were considered, by comparing the constitutive transcriptomic profiles of VAL under healthy and *Ca*Las-infected psyllid infestation.

### Activation of Defense Mechanisms Repressed in VAL Under *Ca*Las-Infected Psyllid Feeding at 1 dpi

Active host defense against pathogen attacks depends on a well-coordinated series of molecular, cellular, and tissue-based mechanisms that follow complex exchanges of signals during the initial host-microbe interactions. If defense mechanisms are not properly activated, both in terms of place and time, they will fail to halt the pathogen and the plant will exhibit disease sensitivity.

In this study, VAL under the healthy and *Ca*Las-infected psyllid infestation showed large differences in the amplitude of transcriptomic changes. VAL response to healthy psyllid infestation was induced at a very early stage (1 dpi), with constitutive expression levels of DEGs putatively involved in the defense mechanism (Figure 3; Supplementary Material 2, Supplementary Figure 5; Supplementary Material 1, Supplementary Tables 7–16). Indeed, many genes, coded for putative pattern recognition receptors (RLK, GLR, and TIR-NB-LRR) and dedicated to the recognition of damage-associated molecular patterns (DAMPs), and to some signaling and defense factors (ethylene, GST, APX, AP2/EREBP, WRKY, MYB, and DOF) were preferentially upregulated in VAL under healthy psyllid infestation at both time points; notably, this is unlike most of the phloem feeder insects (i.e., whiteflies and aphids), which mostly activate the SA-signaling pathway (Kempema et al., 2007; Zarate et al., 2007). Here, the response of VAL to healthy psyllid feeding employed the ethylene-mediated pathways as signaling carriers (Supplementary Material 2, Supplementary Figure 5; Supplementary Material 1, Supplementary Table 9). Several key genes involved in ethylene biosynthesis and signal transduction were upregulated at both time points, including a DEG encoding a member of MAP Kinase Kinase family (ciclev10021170m) (Figure 5) on the signaling pathway independently involved in ethylene biosynthesis, an ACS which is the rate-limiting step of ethylene biosynthesis, and EIN3 which is considered the key transcription factor mediating ethylene response (Figure 6). It clearly showed that the ethylene signaling pathway was activated in response to healthy psyllid infestation. Taken together, these findings suggest that the DAMPs from the piercing and sucking by healthy psyllids induced primary hypersensitive response in the local damaged cells of VAL, and systemic signals were rapidly released and amplified *via* ethylene signaling pathways, plant-wide. Previous studies have observed and reviewed the sophisticated mechanisms of the response of the plants to damage and herbivores (Savatin et al., 2014; Hilleary and Gilroy, 2018; Wang et al., 2020).

Unlike VAL under healthy psyllid infestation, VAL under *Ca*Las-infected psyllid infestation showed delayed response to psyllid/*Ca*Las infestation at 1 dpi. Although genes in the cell redox regulatory system, including *GST* and *APX*, and several heat shock protein genes, were upregulated after *Ca*Las-infected psyllid feeding at 1 dpi (Supplementary Material 2, Supplementary Figure 5; Supplementary Material 1, Supplementary Tables 12, 19), several key genes on the pathways, such as signaling, transcription factor, ethylene biosynthesis, and defense protein-related pathways were either downregulated or expressed with no significant difference, followed by a burst of DEGs at 5 dpi (Figures 3–6; Supplementary Material 1, Supplementary Tables 7–11, 13). This indicated that VAL rapidly responded to the psyllid/*Ca*Las infestation at the injured site (local response); however, the systemic response was repressed at 1 dpi and then overreacted at 5 dpi. Interestingly, in VAL under the *Ca*Las-infected psyllid infestation at 1 dpi, hormone-related gene modulation was also mainly found in the ethylene biosynthesis and signaling pathways. Therefore, we initially took ethylene-related pathways as a breakthrough point.

Intense studies have elucidated the signaling pathways from the sensing of ethylene by its receptors to downstream signaling components and further ethylene-responsive genes (Schaller and Kieber, 2002; Alonso and Stepanova, 2004; Guo and Ecker, 2004; Ju and Chang, 2015). All ethylene-regulated responses begin with the elevation of its biosynthesis by the ACS gene, which is under tight regulation (Kende, 1993). Recently, a study elucidated the detailed mechanisms underlying the wounding/pathogen-induced ethylene biosynthesis, in which ACS is the key player (Li et al., 2018). Surprisingly, our present study showed consistent mechanisms on the ethylene-related pathways. In detail, one DEG (ciclev10019970m) encoding ACS was detected in all the samples, which was significantly upregulated in the VAL under healthy psyllid infestation but expressed with no significant difference in VAL under *Ca*Las-infected psyllid infestation at 1 dpi (Figure 6; Supplementary Material 1, Supplementary Table 9). According to the MapMan annotation, this detected DEG was matched with Arabidopsis *ACS1*, which has an extended C terminal domain that encompasses three mitogen-activated protein kinase (MAPK) phosphorylation sites and a putative calcium-dependent protein kinase (CPK) phosphorylation site (Liu and Zhang, 2004; Kamiyoshihara et al., 2010). It has also been proved that ACS2 is not only regulated transcriptionally but also post-transcriptionally by MAPK and CPK for its stability and function (Kamiyoshihara et al., 2010). Indeed, a DEG (ciclev10021170m) encoding a member of MAP Kinase Kinase family, which independently involved in ethylene biosynthesis by inducing *ACS, ACO, ETR, ERS*, and *ERF*, was significantly upregulated in VAL under the healthy psyllid infestation but expressed with no difference in VAL under the *Ca*Las-infected psyllid infestation at 1 dpi. Moreover, on the ethylene signaling pathway, ethylene is perceived by a family of receptors (ERS1, ETR1, ETR2, EIN4, and ERS2 in Arabidopsis) localized in the endoplasmic reticulum membrane (Deslauriers et al., 2015). Upon binding, ethylene inactivates them and thereby blocks the serine-threonine protein kinase constitutive triple response 1 (CTR1) activity promoting the cleavage of ER-anchored EIN2 protein (Chang, 2016; Hu et al., 2017a), which results in EIN2 C terminus being cleaved and localizing to the nucleus, indirectly triggering EIN3 and EIN3-like (EIL) transcription factors (TFs) that are considered the key transcriptional regulators of ethylene response. The protein encoded by *CTR1* acts as a negative regulator of ethylene signaling (Huang et al., 2003). In our present study, according to the GO annotation (Figure 6; Supplementary Material 1, Supplementary Table 10), one *CTR1* (ciclev10030801m) was downregulated at 1 dpi (−2.16) and upregulated at 5 dpi (+3.82) in VAL under the healthy psyllid infestation, and two *CTR1* (ciclev10030799m, ciclev10030801m) were downregulated in VAL under the *Ca*Las-infected psyllid infestation at 1 dpi. Thereafter, the expression of *EIN3* was supposed to be significantly triggered. Indeed, one DEG (ciclev10000619m) encoding *EIN3* was upregulated in VAL under the healthy psyllid infestation at 1 dpi; however, one DEG (ciclev10007708m) encoding EIN3-binding F box protein 1 was downregulated, and several DEGs encoding *EIN3* were detected to express with no significant difference in VAL under the *Ca*Las-infected psyllid infestation at 1 dpi. Furthermore, as a key transcriptional regulator of ethylene response, EIN3/EIL1 modulates multiple transcriptional cascades. EIN3/EIL1 target genes encoding TFs include *ERF1*, involved in a range of ethylene responses (Solano et al., 1998). Indeed, at 1 dpi, all five identified ERF DEGs were upregulated in VAL under the healthy psyllid infestation, while four and two DEGs encoding *ERF* were upregulated and downregulated in VAL under the *Ca*Las-infected psyllid infestation, respectively (Figure 6; Supplementary Material 1, Supplementary Table 9). Numerous TF-encoding genes comprising representatives of *WRKY, AP2*/*ERF, NAC*, and other families were retrieved from whole-genome data on *EIN3/EIL1* binding and ethylene-induced transcriptomes (Chang et al., 2013). Indeed, four DEGs encoding *AP2/ERF*, five DEGs encoding *MYB*, 11 DEGs encoding *WRKY* were upregulated in VAL under the healthy psyllid infestation, while three DEGs encoding *AP2/ERF*, four DEGs encoding *MYB*, and one DEG encoding *WRKY* were upregulated, and four DEGs encoding *DOF*, three DEGs encoding *MYB* were downregulated in VAL under the *Ca*Las-infected psyllid infestation at 1 dpi. Taken together, VAL rapidly responded to healthy psyllid infestation *via* MAPKs-ACS-ethylene-TFs module; however, several key genes in this module were repressed in VAL under *Ca*Las-infected psyllid infestation at 1 dpi, including a member from the upstream MAPK-signaling pathway.

The MAPK cascades, that function downstream of receptors/sensors and transducer signals from extracellular stimuli to intracellular responses, are highly conserved signaling pathways across eukaryotes (Tena et al., 2011). Wounding-induced transient ethylene biosynthesis has been known for decades; a recent study has shown two independent MAPK modules activated by wounding and insect feeding which regulate notably ethylene production (Sozen et al., 2020). Previous studies have demonstrated that RLKs and RLPs, localized in the plasma membrane are frontiers of the plant immune system. They perceive MAMPs/DAMPs at plant cell surfaces and transmit the immune signals to initiate defense cascades, including ROS, influx of calcium, and activation of MAPKs (Wang et al., 2020). Indeed, 13 DEGs encoding RLKs and one DEG encoding RLP were modulated in VAL under healthy and *Ca*Las-infected psyllid infestation at 1 dpi, and they were matched with seven *RLKs* homolog and one *RLP* in Arabidopsis in MapMan analysis (Table 3). A recent study constructed an LRR-based cell surface interaction network (CSI^LRR^), which analyzed the interactions of 200 leucine-rich repeat receptor-like protein kinases (LRR-RLKs) from Arabidopsis and defined them into four subnetworks (Smakowska-Luzan et al., 2018). We used the seven Arabidopsis RLK homologs in the CSI^LRR^ to detect their interactions with other LRR-RLKs (Supplementary Material 2, Supplementary Figure 6; Supplementary Material 1, Supplementary Table 20). The seven Arabidopsis RLK homologs were distributed into four subnetworks in CSI^LRR^ and had interactions with LRR-RLKs from different subcellular localizations, which indicated that their modulation could rapidly lead to a series of immune responses cell-wide. Interestingly, brassinosteroid insensitive 1-associated receptor kinase 1 (BAK1) (AT4g33430) and APEX (AT5G63710), the two most interconnected and important nodes from two different subnetworks in CSI^LRR^, were found to interact with five of the 13 DEGs; however, none of their citrus homologs (ciclev10001146m, ciclev10002748m, and ciclev10025254m) were identified in the DEG in VAL under both treatments at 1 dpi, but ciclev10002748m (citrus homolog of *BAK1*) and ciclev10025254m (citrus homolog of *APEX*) were significantly upregulated and downregulated, respectively, in VAL under the *Ca*Las-infected psyllid infestation at 5 dpi. Moreover, the Arabidopsis RLK902, which was shown to localize at the plasma membrane and plays an essential role in resistance to the bacterial pathogen *P. syringae* (Zhao et al., 2019), was also detected in our study. Interestingly, three citrus homologs (ciclev10007673m, ciclev10007812m, and ciclev10011289m) of *AtRLK902* were not detected in VAL under both treatments at 1 dpi, but significantly downregulated in VAL under *Ca*Las-infected psyllid infestation at 5 dpi. Taken together, we speculate that the proteins encoded by these modulated *RLKs* played important roles in perceiving MAMPs/DAMPs and initiating downstream signaling transduction (i.e., MAPK signaling pathways) in VAL under both treatments. Especially, those modulated in VAL under *Ca*Las-infected psyllid infestation had interactions with several other important RLKs, which might be the initial cause for the signaling repression at 1 dpi and overactive defense responses at 5 dpi.

**Table 3:** The modulated RLKs/RLP identified in VAL under healthy and *Ca*Las-infected psyllid infestation at 1 dpi.

**ID**	**Healthy_psyllid_1dpi**	***Ca*Las-infected_psyllid_1dpi**	**Best-hit-Arabi-name**	**Arabi-defline**
ciclev10000513m	7.181193	7.828045	AT5G58300.2	Leucine-rich repeat protein kinase family protein
ciclev10031027m	−3.539732		AT4G18640.1	Leucine-rich repeat protein kinase family protein
ciclev10013722m		−2.3637984	AT1G71400.1	receptor like protein 12
ciclev10014427m	−4.127454	−5.6762094	AT1G07650.2	Leucine-rich repeat transmembrane protein kinase
ciclev10014641m		−1.1596398	AT1G56140.1	Leucine-rich repeat transmembrane protein kinase
ciclev10020580m	3.8825603		AT2G31880.1	Leucine-rich repeat protein kinase family protein
ciclev10008474m	1.8380265		AT4G08850.1	Leucine-rich repeat receptor-like protein kinase family protein
ciclev10013898m	4.7302938		AT4G08850.1	Leucine-rich repeat receptor-like protein kinase family protein
ciclev10018554m	3.9241092		AT4G08850.1	Leucine-rich repeat receptor-like protein kinase family protein
ciclev10014659m	4.661711		AT4G08850.1	Leucine-rich repeat receptor-like protein kinase family protein
ciclev10014216m	4.8468714		AT4G08850.1	Leucine-rich repeat receptor-like protein kinase family protein
ciclev10018687m	2.9997098		AT3G47570.1	Leucine-rich repeat protein kinase family protein
ciclev10013537m		−2.69563	AT3G47570.1	Leucine-rich repeat protein kinase family protein
ciclev10027688m		1.7315834	AT3G47570.1	Leucine-rich repeat protein kinase family protein

The interaction analysis of RLK DEGs did not show the detailed underlying mechanism(s) of the signaling repression; however, we observed a DEG (ciclev10014641m) encoding RLK and a DEG (ciclev10013722m) encoding RLP were significantly downregulated in VAL under *Ca*Las-infected psyllid infestation at both time points but not identified as DEG in VAL under the healthy psyllid infestation. Interestingly, this *RLK* was found to have a downregulated pattern in VAL (sensitive variety) and an upregulated pattern in rough lemon (tolerant variety) at four time points (0, 7, 17, and 34 weeks after grafting inoculation) in another study (unpublished data), which raised our interest to seek its role in plant defense. A study on the Arabidopsis oomycete downy mildew pathogen found that marked differences were observed between early and late stages of infection, but a gene encoding a LRR-RLK was constantly upregulated, and a knockout mutant showed reduced pathogen infection (Hok et al., 2011). Similarly, comparing the present study with several previous transcriptomic studies on *Ca*Las-infected citrus plants (Albrecht and Bowman, 2008; Liao and Burns, 2012; Martinelli et al., 2012; Aritua et al., 2013; Xu et al., 2015; Wang et al., 2016; Arce-Leal et al., 2020), significant differences were observed between early and late stages of infection. It is speculated that this *RLK* (ciclev10014641m) plays a crucial role in *Ca*Las and citrus interaction. Previous studies showed that ligand binding presumably induces the dimerization or oligomerization of RLKs, either with themselves or with a co-receptor, leading to the activation of intracellular kinase domains to initiate downstream signaling transduction and regulate multiple biological functions (Schlessinger, 2002; Lemmon and Schlessinger, 2010; Bredow and Monaghan, 2019). We expected to find the co-receptor(s) of this RLK (ciclev10014641) *via* protein-protein interaction analysis; however, no putative DEG was found to interact with this receptor kinase at the protein level by the STRING database. Nevertheless, we did further study on *RLP* (ciclev10013722m), which had the similar downregulation pattern in our study on *RLK* (ciclev10014641). According to the MapMan analysis, this identified *RLP* encodes a CLV2-(CLAVATA2) related protein, which has been shown to control cell divisions in the shoot and root apical meristem, vascular, and legume nodules (Ferguson et al., 2014; Matsubayashi, 2014; Gaillochet et al., 2015; Hastwell et al., 2015). Studies have shown that CLV2, in conjunction with the other receptor and RLK, formed a receptor complex to perceive nematode-secreted peptides allowing nematodes to successfully infect Arabidopsis roots (Pan et al., 2016). Moreover, it is well-known that CLV2 encodes a receptor-like protein that lacks a cytoplasmic kinase domain; therefore, it needs to interact with additional component(s), possibly with RLK(s), to activate cellular responses upon ligand perception. The association of CLV2 with different regulatory RLKs might result in the activation of distinct biological responses, implying that the diversity of CLV2-associated receptor complexes partially determine the specificity of CLV2-mediated signaling. So far, potential interacting partners for CLV2 are unknown in most cases (Pan et al., 2016). Taken together, we speculated that the protein encoded by *RLK* (ciclev10014641m) itself, or interacting with the protein encoded by *RLP* (ciclev10013722m), might be repressed by the *Ca*Las-secreted MAMP(s) during *Ca*Las infection in the sensitive cultivar VAL, which resulted in the delayed defense signaling and further downstream gene modulation.

As mentioned above, ACS also has an extended C terminal domain that encompasses a CPK phosphorylation site. Although an early study indicated that MAPK and CPK pathways do not function independently and that a concerted activation of both pathways controls the response specificity to biotic and abiotic stress in plants (Ludwig et al., 2005), a recent study demonstrated that the regulation of Arabidopsis CPK5/6 on ethylene production in response to wounding and *B. cinerea* infection, through the modulation of ethylene biosynthesis enzyme ACS, was independent of the MAPK modules (Li et al., 2018). Indeed, several CPK DEGs were identified in VAL under both treatments at 1 dpi (Figure 5), which might be initiated by the modulation of upstream *RLKs* and *GLRs* (Mousavi et al., 2013; Wang et al., 2020). Interestingly, two DEGs encoding GLR were significantly upregulated in VAL under healthy psyllid infestation, while genes encoding GLR were not modulated in VAL under the *Ca*Las-infected psyllid infestation at 1 dpi (Figure 4). We speculated that the inactivated *GLR* in VAL under the *Ca*Las-infected psyllid infestation at 1 dpi might be caused by the lack of glutamate, which was uptaken and utilized by *Ca*Las (Duan et al., 2009). On the other hand, upon perceiving the MAMPs/DAMPs, the RLKs decode them into signals and transmit them to downstream defense reactions, including the influx of cytosolic Ca^2+^, which further initiate CPK signaling pathways. Studies indicated that CPK family members mediate and transmit defense signals in response to pathogen-associated molecular patterns as well as pathogen effectors (Cheng et al., 2002; Boudsocq and Sheen, 2013; Schulz et al., 2013; Romeis and Herde, 2014). Several studies have indicated that CPK5, as a central hub, plays a unique role in the local and distal immune signaling (Boudsocq et al., 2010; Dubiella et al., 2013). Excitingly, a DEG (ciclev10028596m), annotated by MapMan as *calmodulin-domain protein kinase CDPK isoform 5* (*CPK5*), was detected which was significantly downregulated in VAL under *Ca*Las-infected psyllid infestation at 1 dpi (Figure 5), but significantly upregulated at 5 dpi (Supplementary Material 1, Supplementary Table 7); and this *CPK5* was identified to express with no difference in VAL under the healthy psyllid infestation at both time points. As the result showed, in VAL under *Ca*Las-infected psyllid infestation at 1 dpi, although the detected *CPK5* was significantly downregulated, the expression of *MAPK* (ciclev10021170m) and *ACS*(ciclev10019970m) expressed as in the control, were not affected. Therefore, we speculated that the induced ethylene signaling pathways by CPK5 in response to psyllid/*Ca*Las infestation were independent of the MAPK module in VAL.

In addition to all these modulated DEGs discussed above, one DEG (ciclev10019103m) encoding a RelA-SpoT homolog (RSH) was significantly downregulated in VAL under the *Ca*Las-infected psyllid infestation at 1 dpi. Studies have identified RelA/SpoT and their homologs, RSH involved in stringent response in bacteria and plants (Cashel and Gallant, 1969; Ito et al., 2017). Further study of their product (p)ppGpp-guanosine tetra-and pentaphosphates in Arabidopsis found that ppGpp-dependent regulatory mechanisms exist in plastids and contribute to the fine-tuning of chloroplast development and metabolism, and the control of chloroplast development by ppGpp is one of the most important events for plastidial stringent response (Maekawa et al., 2015). In our present study, the PS pathways were severely downregulated in VAL under the *Ca*Las-infected psyllid infestation at 5 dpi. Several previous transcriptomic studies observed the similar phenomenon; however, a study with Kaffir lime (a tolerant cultivar) found that the PS process was not disturbed by *Ca*Las infection (Hu et al., 2017b). With limited knowledge, the regulation mechanism underlying this downregulated *RSH* in VAL is unknown. We speculated that this *RSH* might play a significant role in the downregulation of chloroplast metabolism in VAL.

### An Overactive but Overwhelmed Passive Defense Reaction in VAL Under *Ca*Las-Infected Psyllid Infestation at 5 dpi

At 5 dpi, a burst of DEG was identified in VAL under *Ca*Las-infected psyllid infestation. According to the PageMan analysis, many bins were enriched in all functional categories in VAL under *Ca*Las-infected psyllid infestation (Figure 3). All the bins on PS pathways were severely downregulated, as we discussed above that a DEG (ciclev10019103m) encoding RSH might play a key role in the decreased photosynthesis. Another notable finding was that the ATP biosynthesis pathway was significantly upregulated in VAL under *Ca*Las-infected psyllid infestation but rarely affected in VAL under the healthy psyllid infestation, which indicated a strong demand for ATP in *Ca*Las-infected VAL, and raised the question of whether *Ca*Las scavenged ATP from VAL for its rapid replication. Indeed, according to the genome analysis, *Ca*Las encodes for an ATP/ADP translocase in addition to its ATP synthase, allowing it to both synthesize ATP as well as uptake this energy source directly from its surroundings (Duan et al., 2009). Forty ABC transporters, which were energized by ATP hydrolysis, were also identified in the *Ca*Las genome, which is much more than the average number (15) in the intracellular bacteria of a similar size (Davidson et al., 2008; Duan et al., 2009). Moreover, as a phloem limited pathogen, *Ca*Las was injected into the phloem cells by psyllid feeding, and then replicated mainly in the sieve elements. Considering the special cell structure of the sieve elements, which lack a nucleus and have very few organelles at maturity, we speculated that *Ca*Las could initially take over the glycolysis in the cytosol for ATP production. Very interestingly, a recent study on several viruses found that the viruses hijacked the cellular glycolytic and fermentation pathways to rapidly produce ATP locally for its replication. They also found that the knockdown of *Pdc1 pyruvate decarboxylase* and *Adh1 alcohol dehydrogenase* fermentation enzymes in plants greatly reduced the efficiency of tombusvirus replication, and enzymatically functional Pdc1 is required to support tombusvirus replication (Lin et al., 2019; Nagy and Lin, 2020). Surprisingly, both the Pdc1 and Adh1 were detected in our present study (Supplementary Material 1, Supplementary Table 21). In VAL under *Ca*Las-infected psyllid infestation, one DEG (ciclev10011649m) encoding Pdc1 was upregulated at 1 dpi, while three DEGs encoding Adh1 were downregulated but several other DEGs on the glycolytic pathway were upregulated at 5 dpi. Moreover, an *in silico* analytical study of the *Ca*Las genome has found that a key glycolytic gene, glyoxalase I (*gloA*), was missing, which indicated that there was a dysfunction of the glycolysis pathway in *Ca*Las itself (Jain et al., 2017). As a consequence of the less activated (three upregulated DEGs) ATP biosynthesis pathway in mitochondria at 1 dpi, but strongly activated pathway (18 upregulated DEGs) at 5 dpi, we speculate that *Ca*Las might initially take over the glycolytic pathway in the sieve elements for the ATP and molecular building blocks production at 1 dpi, and then manipulate the glycolytic pathways and tricarboxylic acid cycle and oxidative phosphorylation pathways for more plentiful ATP. Meanwhile, a large number (369) of DEGs involved in response to biotic stress were identified as requiring ATP/GDP/GTP and phosphate for function (Figure 8; Supplementary Material 1, Supplementary Table 18). The number of these defense-related DEGs in VAL under *Ca*Las-infected psyllid infestation was significantly higher than that under healthy psyllid infestation at 5 dpi. Especially, the upregulation of DEGs (108) on the hormone pathway indicated that VAL (sensitive host) did initiate defense responses to *Ca*Las infection, which was also found in a previous study of another sensitive citrus cultivar, Cleopatra (Albrecht and Bowman, 2012). However, the large number of downregulated DEGs indicated there was insufficient ATP/GDP/GTP or phosphate, which suggested there might be an energy competition between VAL and *Ca*Las. Interestingly, previous studies on both host and vector of *Ca*Las have suggested that *Ca*Las might alter the host/vector environment to enhance the nutrient availability and increase the ATP levels (Killiny et al., 2017; Lu and Killiny, 2017; Pitino et al., 2017), which is consistent with our present study.

It is noteworthy that several DEGs on JA and SA pathways were upregulated, and a large number of DEGs on ABA, AUX, and BR pathways were downregulated in VAL under the *Ca*Las-infected psyllid infestation at 5 dpi, which were not detected in VAL under the healthy psyllid infestation at both time points. Previous transcriptomic studies in *Ca*Las-infected citrus have shown the modulated ethylene, JA, and SA pathways in leaves and fruits with different patterns (Martinelli et al., 2012; Xu et al., 2015), which might be associated with different tissues and sample times. In our present study, a large number of DEGs involved in the signaling cascade, such as the *RLKs, MAPKs*, and *CPKs* were activated in VAL under the *Ca*Las-infected psyllid infestation at 5 dpi, which might interconnect with those phytohormones, and the phytohormones might crosstalk with each other. These signaling phytohormones then interacted with the transcription factors, resulting in the regulation of transcription activity and gene expression. Therefore, it is not surprising to identify an abundance (241) of DEGs on the secondary metabolism pathways even at the very early stage (5 dpi). Several key genes on the secondary metabolism pathways were modulated, including the upregulated GGPP synthase, CAOMT, cytochrome P450, and downregulated *CCoAOMT, PAL, DXS, DXR, HDR*, ten DEGs involved in carotenoid biosynthesis, and three DEGs involved in terpenoid biosynthesis. Especially, studies have found that plugged phloem sieve tubes are one of the typical symptoms in *Ca*Las-infected citrus, which appears to be a primary means of defense against *Ca*Las (Trivedi et al., 2009; Bendix and Lewis, 2018). Callose deposition at sieve plates and companion cell plasmodesmata is believed to be an important phloem-localized response to wounding and pathogens (Hao et al., 2008; Millet et al., 2010). This finding was also detected in our present study (Supplementary Material 1, Supplementary Table 22), a callose synthase (ciclev10030476m) was upregulated in VAL under both treatments at 5 dpi, and three glucan synthase-like DEGs were downregulated in VAL under *Ca*Las-infected psyllid infestation. Moreover, although the true physiological role of phloem-localized proteins remains unclear, P proteins are also thought to rapidly seal plates after damage (Batailler et al., 2012; Ernst et al., 2012). Very interestingly, at 1 dpi, three *PP2* were upregulated in VAL under the healthy psyllid infestation but no *PP2* was identified in VAL under the *Ca*Las-infected psyllid infestation; however, at 5 dpi, another three *PP2* were upregulated in VAL under the *Ca*Las-infected psyllid infestation but no *PP2* was identified in VAL under the healthy psyllid infestation (Supplementary Material 1, Supplementary Table 23). A previous study reported the same result that callose deposition is a slower process than P protein accumulation at sieve plates (Hao et al., 2008; Luna et al., 2011). These findings indicated that in response to psyllid feeding, the P proteins were induced in VAL earlier than callose, which might play an important role in the sieve tube plugging mechanism. However, this response was repressed at the very beginning in VAL under the co-occurring attacks by psyllid and *Ca*Las. This finding is consistent with previous reports that have observed that sensitive varieties establish callose defenses too slowly to prevent pathogen spread to the roots, and the colonization in the roots may lead to a reservoir of pathogens that can no longer be controlled by plant defense processes (Johnson et al., 2014). Furthermore, the sieve plates plugging, together with psyllid/CaLas feeding, is believed to cause significant changes of the source-sink system in the citrus plant (Huot et al., 2014). Excessive starch accumulation in the phloem cells is one typical result of altered source-sink allocations. In our present study, at 1 dpi, very few starch metabolism-related DEGs were identified in both treatments; at 5 dpi, few DEGs related to sucrose metabolism pathways were detected in VAL under the healthy psyllid infestation, while many DEGs involved in starch and sucrose metabolism pathways in VAL under the *Ca*Las-infected psyllid infestation was identified (Supplementary Material 1, Supplementary Table 21). Starch and sucrose are coordinately regulated in plants. According to the KEGG pathway analysis, surprisingly, the key regulatory enzyme in starch synthesis, ADP glucose pyrophosphorylase, was significantly downregulated in VAL under the *Ca*Las-infected psyllid infestation, as well as two starch branching enzyme DEGs; however, several DEGs encoding starch degradation enzyme were upregulated, such as one alpha-amylase, four glycosyl hydrolase, as well as one sucrose synthase. The result indicated that the starch accumulation was not initiated at the very beginning (5 dpi). According to the MapMan pathway analysis, we visually observed that most of the DEGs involved in the starch metabolism pathways were downregulated, while several DEGs involved in the sucrose metabolism were upregulated (Supplementary Material 2, Supplementary Figure 7). This finding further supports our hypothesis suggested above, that *Ca*Las might manipulate the glycolytic pathway for ATP production *via* sucrose metabolism and starch degradation. It is not a surprise that our finding is different from the observation of previous studies that excessive starch accumulated in the phloem sieve elements, because our observation is at the very beginning of the infection process and source-sink allocations development.

## Conclusions

Taken together, at 1 dpi, the defense response to healthy psyllid piercing and sucking was successfully activated in VAL *via* RLKs/GLRs-MAPKs-ethylene signaling-mediated systemic immunity. However, the defense response to *Ca*Las-infected psyllid infestation was repressed in VAL. We speculated that a constantly downregulated DEG (ciclev10014641) encoding RLK was repressed by *Ca*Las secreted effector(s), and/or in conjunction with an RLP encoded by a DEG (ciclev10013722m), which might play a crucial role in the downstream signal transduction. Indeed, a DEG (ciclev10021170m) from the MAPK signaling pathway and a DEG (ciclev10028596m) from the CPK signaling pathway were repressed, which further led to the repressed ethylene signaling pathway during the co-occurring attacks by psyllid and *Ca*Las. At 5 dpi, VAL responded to the healthy psyllid infestation with a proper mechanism. However, a burst of DEGs were identified in VAL in response to the *Ca*Las-infected psyllid infestation. The downregulated DEG encoding RSH at 1 dpi might play a key role in the severely downregulated photosynthetic pathways. The severely disturbed signaling cascade orchestrated the biogenesis downstream, in which the upregulated ATP biosynthesis might be demanded by both plant defense metabolism and *Ca*Las rapid replication, which further led to an overwhelmed and passive defense reaction in VAL. Moreover, in VAL under the healthy psyllid infestation, DEGs involved in P protein biosynthesis (1 dpi) were induced earlier than DEGs involved in callose synthesis (5 dpi). However, in VAL under *Ca*Las-infected psyllid infestation, DEGs involved in P protein biosynthesis and callose synthesis were both induced at 5 dpi, which indicated that the sieve tube plugging defense mechanism was repressed by co-occurring attacks by psyllid and *Ca*Las. Furthermore, *Ca*Las might manipulate the glycolytic pathway for ATP and molecular building blocks production *via* starch degradation and sucrose metabolism.

**Figure 9:**
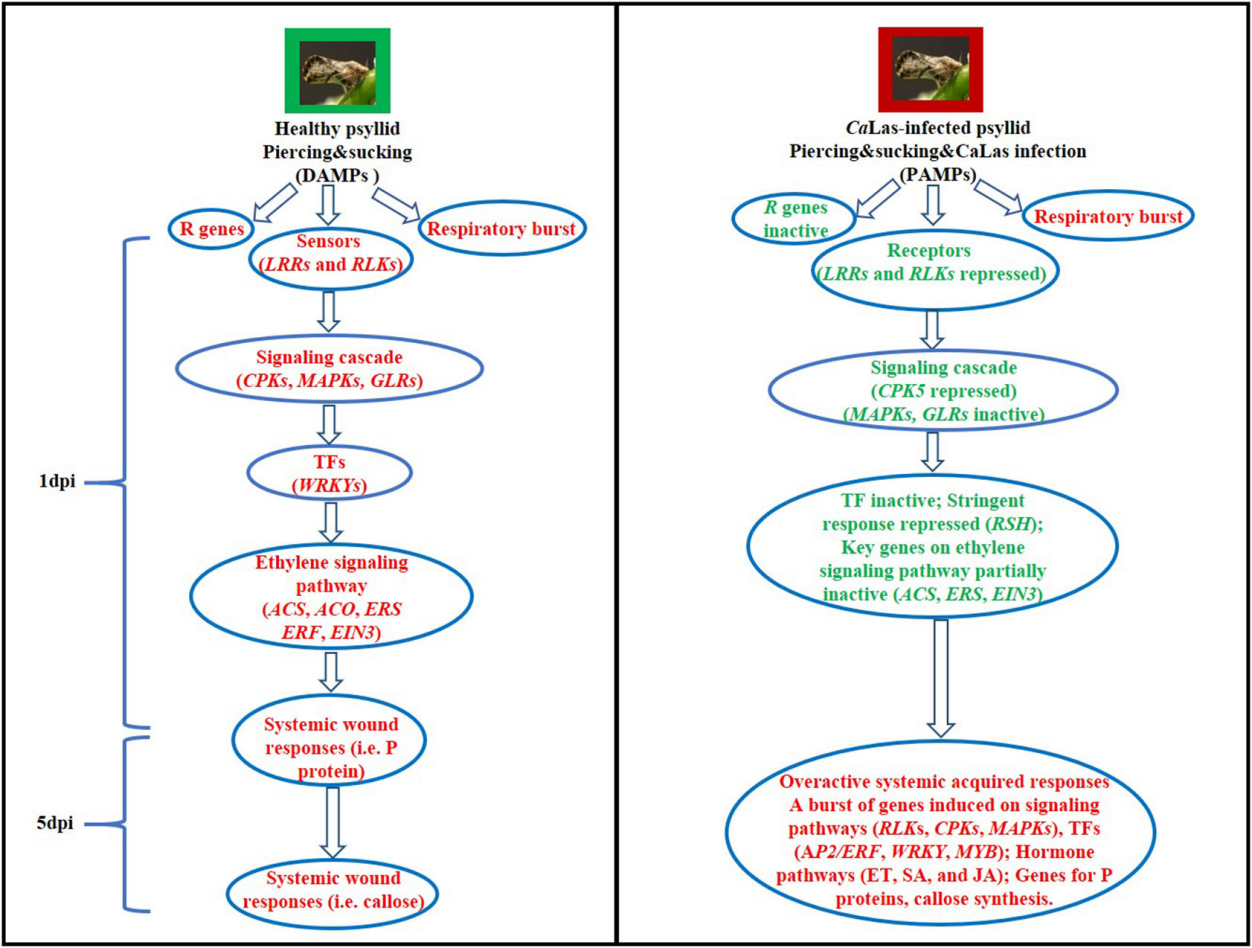
Hypothetical models of the defense mechanisms in VAL under healthy and *Ca*Las-infected psyllid infestation at 1 and 5 dpi. Words in red represent activated steps and words in green represent repressed steps.

Collectively, the data reported in this work indicated that *Ca*Las secreted signals and strongly demanded energy caused the repression of signaling cascade and led to the delayed defense response to the co-occurring attacks by psyllid and *Ca*Las (Figure 9). We speculated that *Ca*Las might have employed virulence strategies to subvert the cell immunity-cell surface receptor and kinases, CPKs and MAPKs, and hormone signaling, and then to manipulate the cell machinery-ATP biosynthesis pathways for energy and molecular building blocks in VAL. Several key genes on those disturbed pathways were addressed, and in-depth further studies on the detailed mechanisms of susceptibility of VAL to *Ca*Las are necessary to confirm these suggestions and to find out feasible solutions in view of a more sustainable citrus culture.

## Data Availability Statement

The RNA-seq raw data of all samples were deposited in the NCBI Sequence Read Archive with accession number SRP271181. The file of gene expression levels was deposited in the NCBI Gene Expression Omnibus (GEO) with accession number GSE15415.

## Author Contributions

FG and QBY conceived and designed the experiments. AM performed the experiments, library construction, and sequencing. XW analyzed the data and wrote the paper. FG, QBY, and XW edited the paper. All authors contributed to the article and approved the submitted version.

## Conflict of Interest

The authors declare that the research was conducted in the absence of any commercial or financial relationships that could be construed as a potential conflict of interest.
