# Supplementary material for: The Mechanism of Citrus Host Defense Response Repression at Early Stages of Infection by Feeding of Diaphorina citri Transmitting Candidatus Liberibacter asiaticus
Source: Front Plant Sci. 2021 Jun 8;12:635153. doi: 10.3389/fpls.2021.635153 (PMC8218908; doi:10.3389/fpls.2021.635153)
Supplement: Supplementary file 1 [file Data_Sheet_1.PDF]

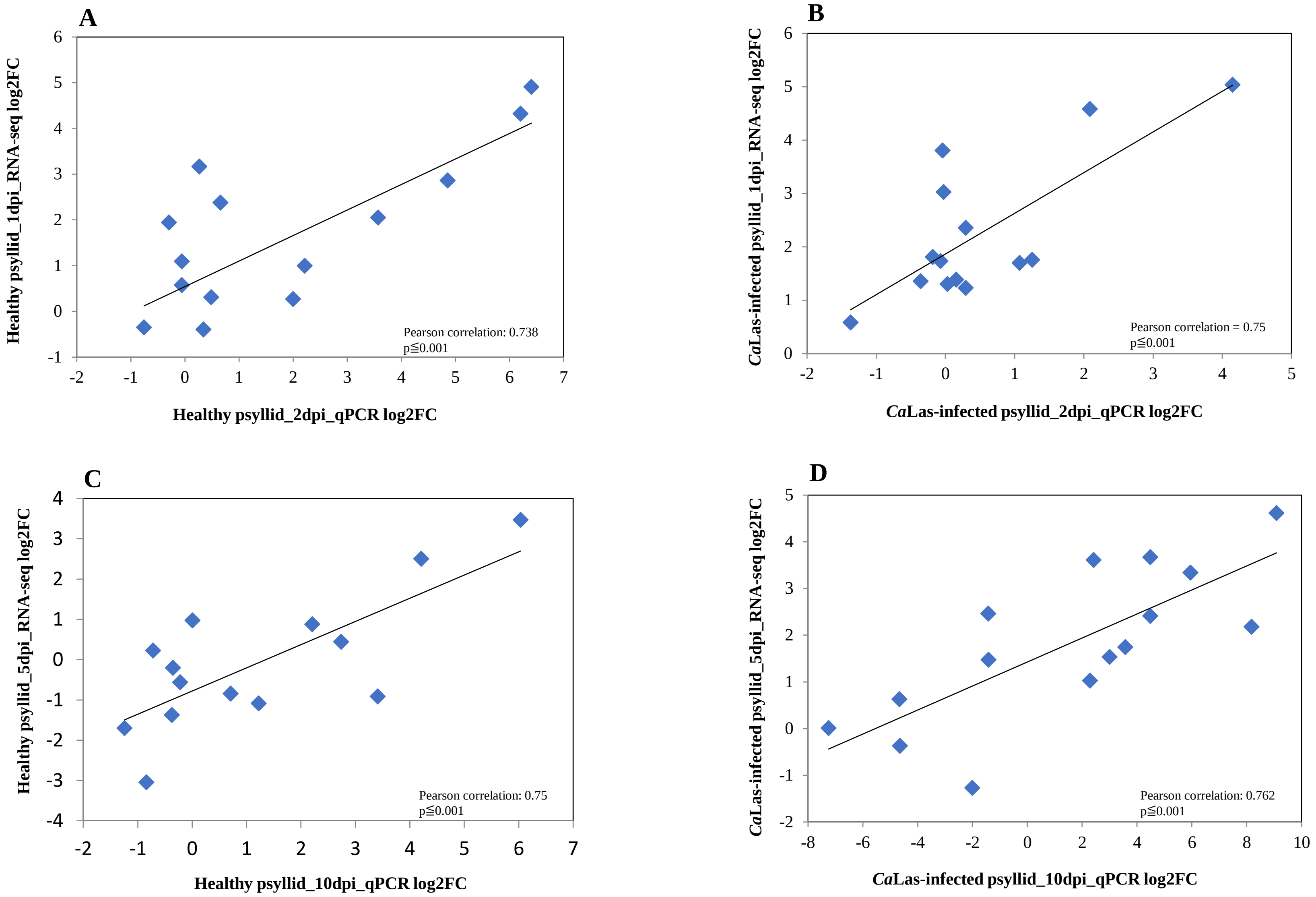

Figure S1. Correlation of 15 selected DEGs gene expression (log2FC) between RNA-seq and qPCR validation in four pairwise comparisons. A: Healthy psyllid_1dpi RNA-seq vs Healthy psyllid_2dpi qPCR; B: *Ca*Las-infected psyllid_1dpi RNA-seq vs *Ca*Las-infected psyllid_2dpi qPCR; C: Healthy psyllid_5dpi RNA-seq vs Healthy psyllid_10dpi qPCR; D: *Ca*Las-infected psyllid_5dpi RNA-seq vs *Ca*Las-infected psyllid_10 dpi qPCR.

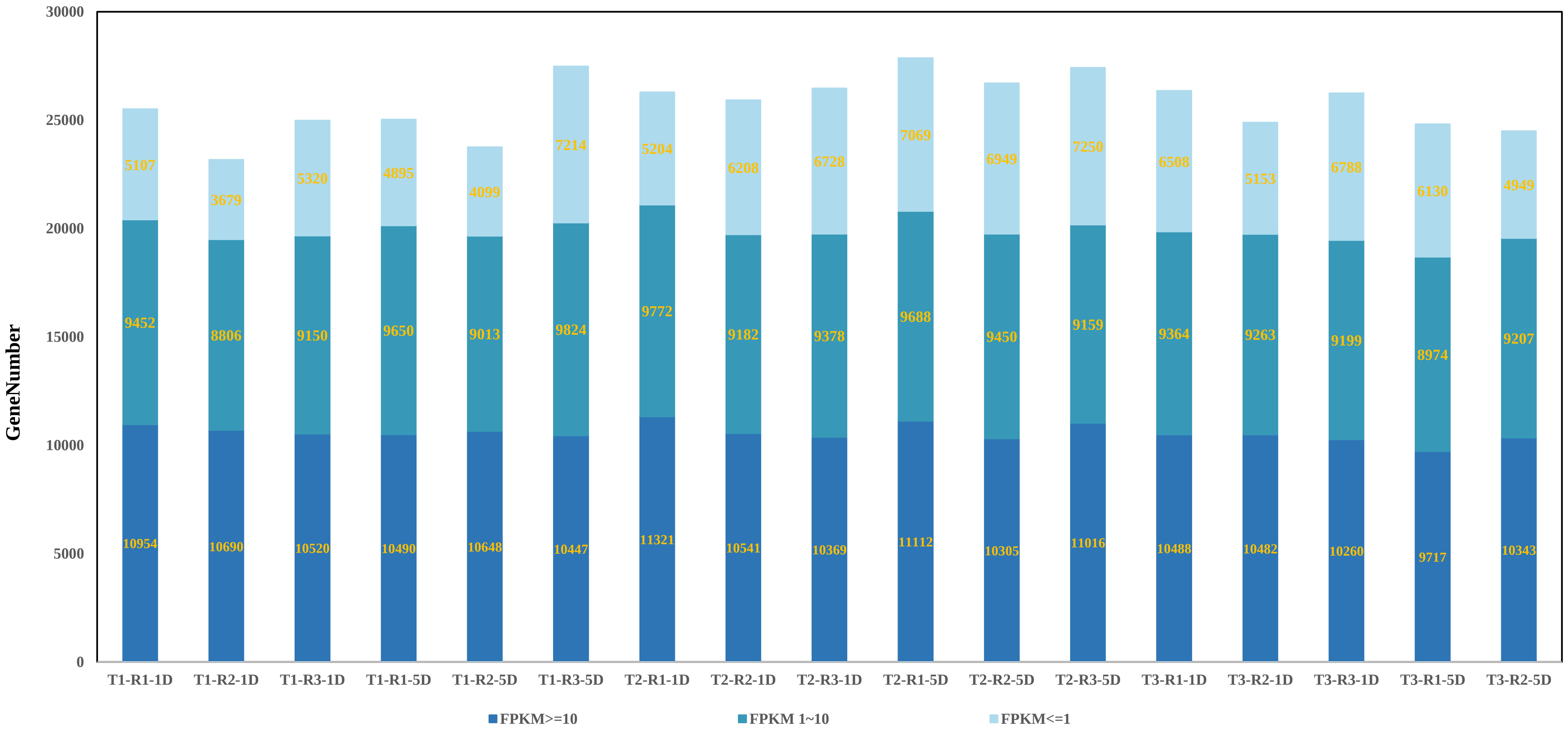

Figure S2. Quantification of transcript expression levels. T1: no psyllid treatment; T2: healthy psyllid treatment; T3: *Ca*Las-infected psyllid treatment; R: replicate; D: day after infestation; FPKM: Fragments Per Kilobase of Transcript per Million mapped reads. Dark blue, FPKM>=10; blue FPKM 1~10; light blue FPKM<=1.

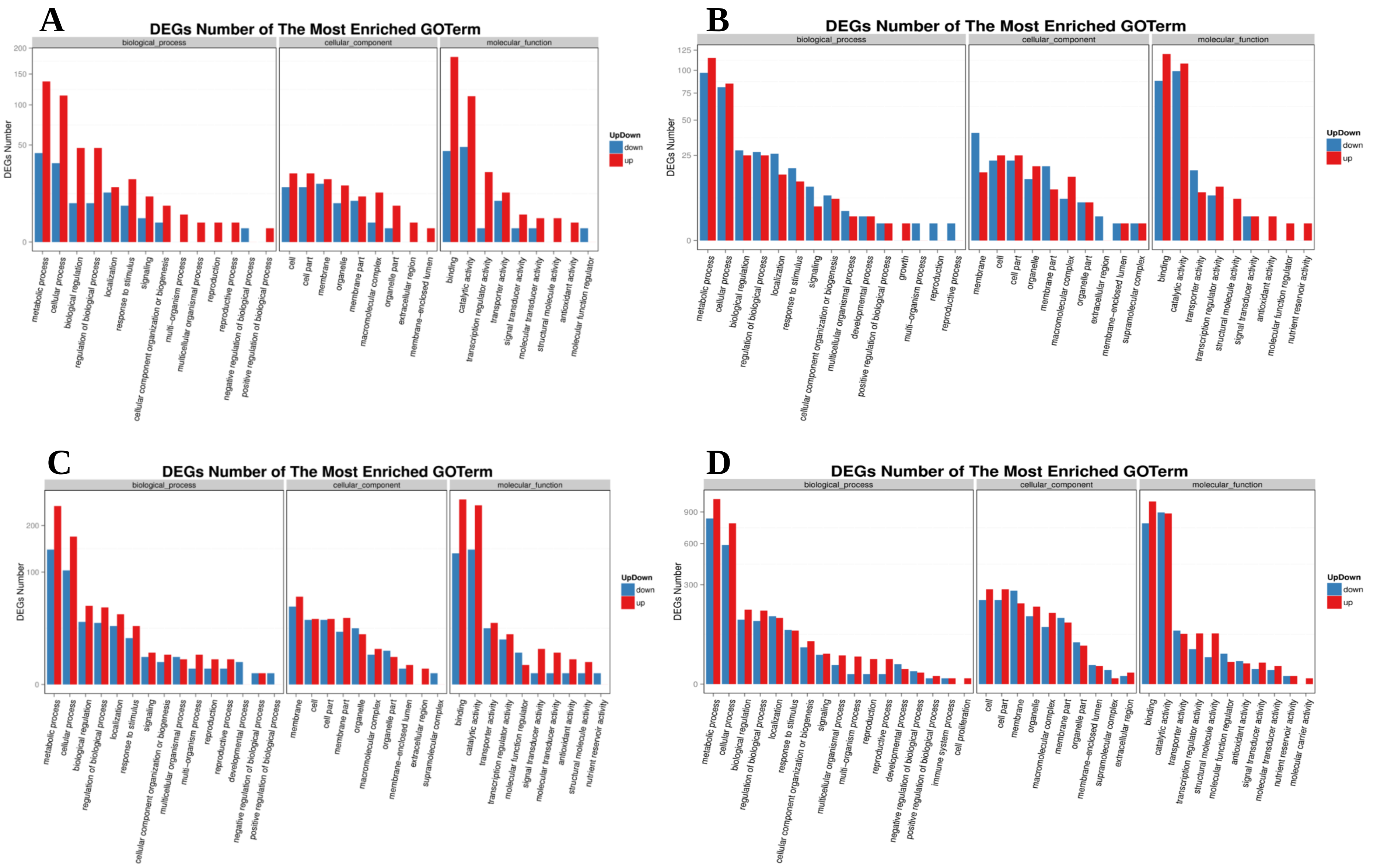

Figure S3. GO analysis of DEGs after healthy and *Ca*Las-infected psyllid infestation in Valencia sweet orange at 1 and 5 days, respectively. (A) DEGs induced by healthy psyllid at 1 dpi. (B) DEGs induced by *Ca*Las-infected psyllid at 1 dpi. (C) DEGs induced by healthy psyllid at 5 dpi. (D) DEGs induced by *Ca*Las-infected psyllid at 5 dpi. Red, upregulated DEGs; blue, downregulated DEGs.

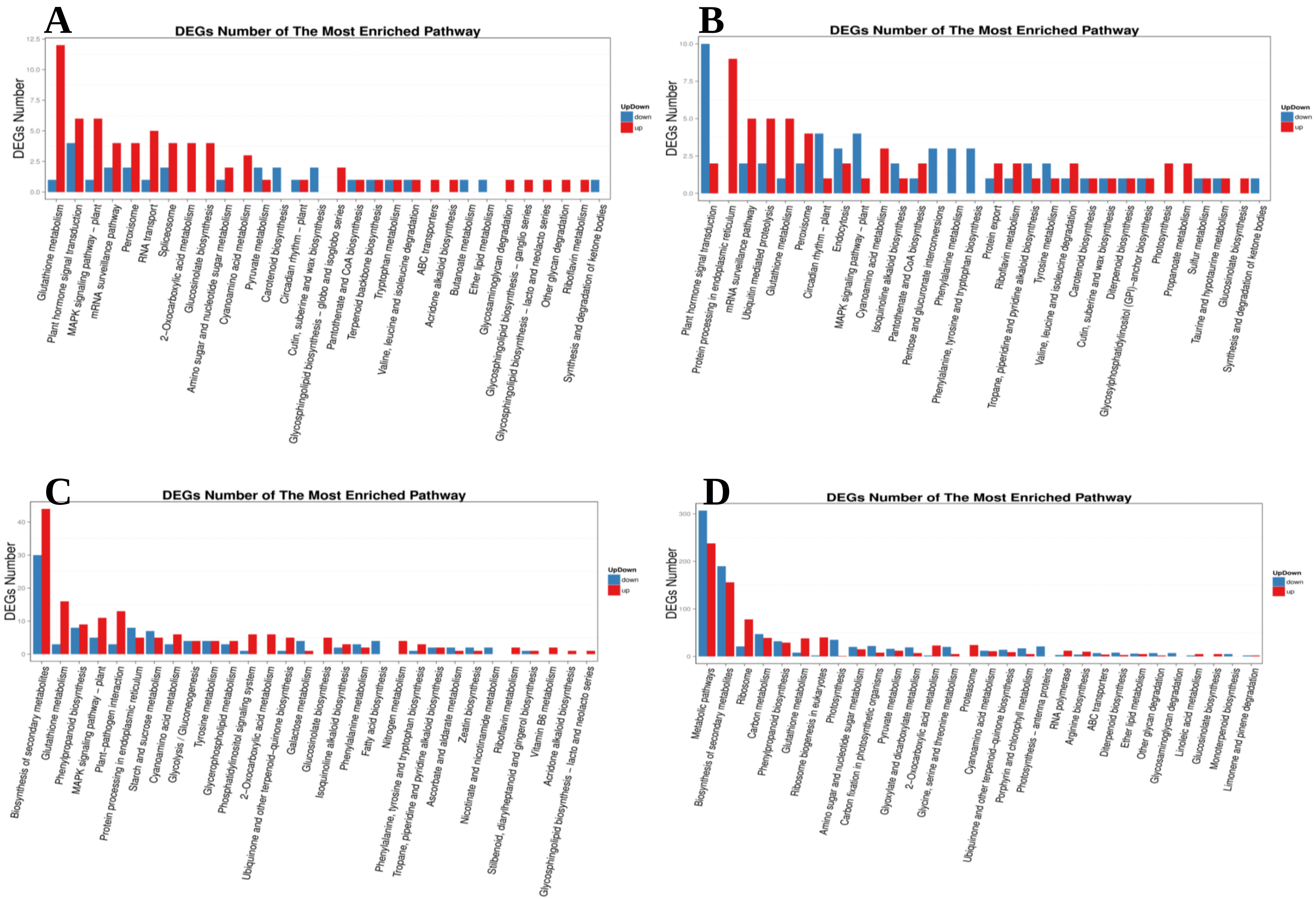

Figure S4. KEGG analysis of DEGs after healthy and infected psyllid infestation in Valencia sweet orange at 1 and 5 days, respectively. (A) DEGs induced by healthy psyllid at 1dpi. (B) DEGs induced by *Ca*Las-infected psyllid at 1 dpi. (C) DEGs induced by healthy psyllid at 5 dpi. (D) DEGs induced by *Ca*Las-infected psyllid at 5 dpi. Red, upregulated DEGs; blue, downregulated DEGs.

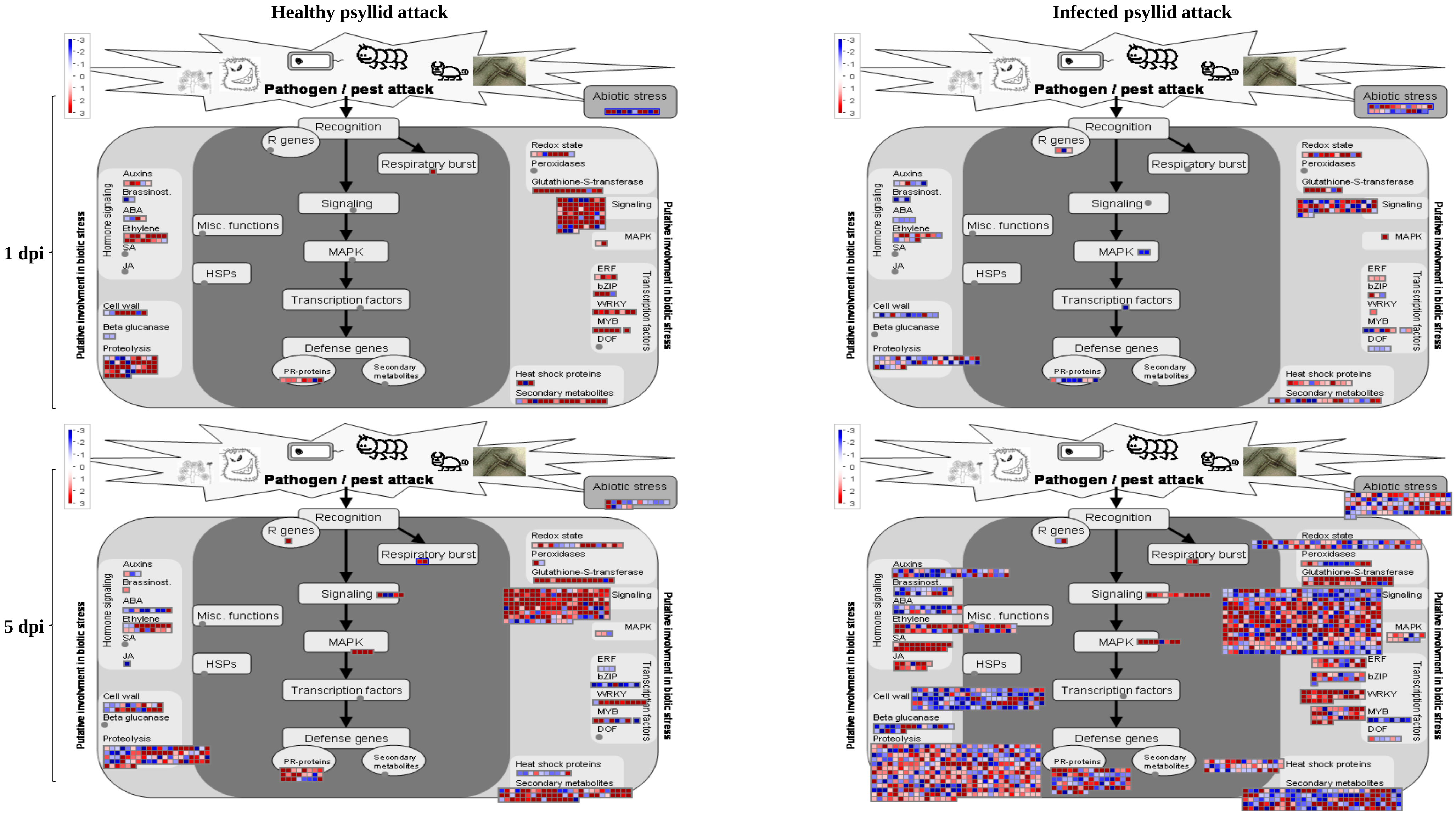

Figure S5. MapMan illustration depicting DEGs from the "Biotic stress" bins affected by healthy and *Ca*Las-infected psyllid infestations in Valencia sweet orange. Transcriptomic data obtained from healthy and *Ca*Las-infected psyllid infestation at 1 and 5 dpi, were compared to their respective controls (no psyllid). Log2 fold changes were indicated as gradient of blue (downregulated) and red (upregulated).

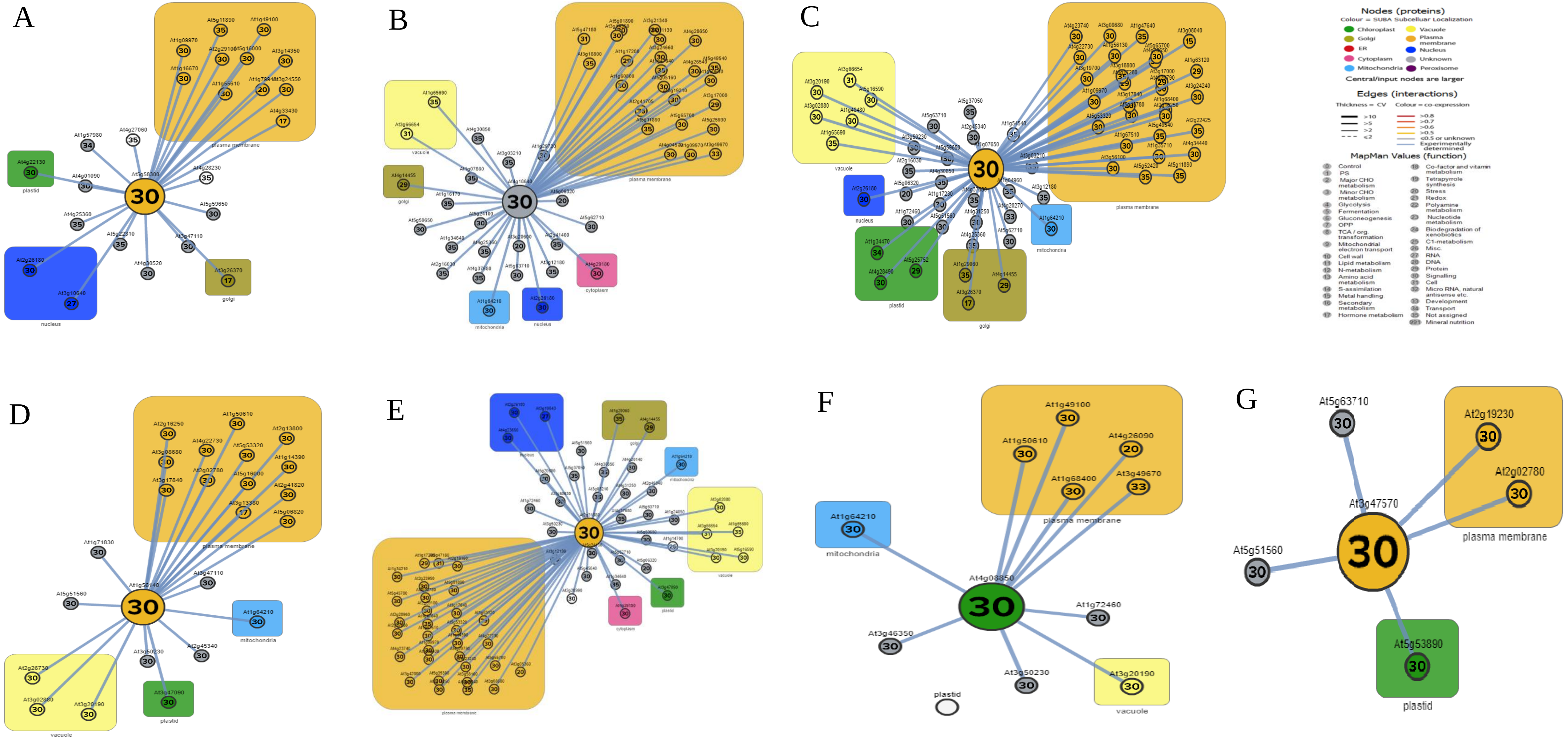

Figure S6. Interaction partners of the 13 modulated DEGs encoding RLKs in VAL under healthy and *Ca*Las-infected psyllid infestation at 1 dpi screened in CSI^{LRR}. A: ciclev10000513m; B: ciclev10031027m; C: ciclev10014427m; D: ciclev10014641m; E: ciclev10020580m; F: ciclev10008474m, ciclev10013898m, ciclev10018554m, ciclev10014659m, ciclev10014216m; G: ciclev10018687m, ciclev10013537m, ciclev10027688m.

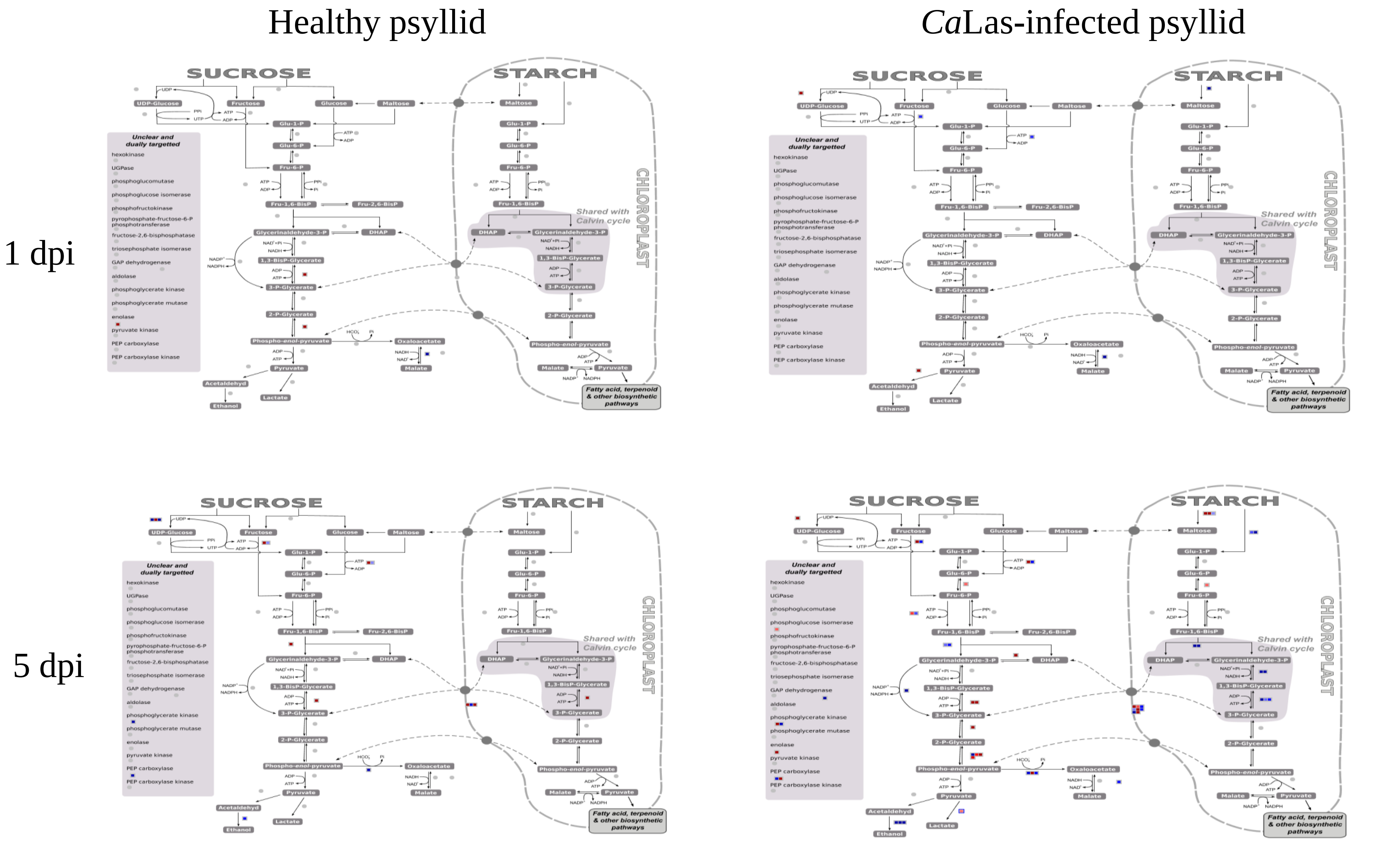

Figure S7. Transcriptional modulation of genes involved in glycolytic pathways after healthy and infected psyllid attacks in Valencia sweet orange at 1 and 5 days, respectively. DEGs colored in red are significantly up-regulated, whereas DEGs colored in blue are significantly down-regulated.
